# Effects of a Novel Thiadiazole Derivative with High Anticancer Activity on Cancer Cell Immunogenic Markers: Mismatch Repair System, PD-L1 Expression, and Tumor Mutation Burden

**DOI:** 10.3390/pharmaceutics13060885

**Published:** 2021-06-15

**Authors:** Sofia Sagredou, Panagiotis Dalezis, Eirini Papadopoulou, Maria Voura, Maria V. Deligiorgi, Michail Nikolaou, Mihalis I. Panayiotidis, George Nasioulas, Vasiliki Sarli, Dimitrios T. Trafalis

**Affiliations:** 1 Laboratory of Pharmacology, Medical School, National & Kapodistrian University of Athens, 75 Mikras Asias Street, 11527 Athens, Greece; pdalezis@med.uoa.gr (P.D.); mdeligiorgi@yahoo.com (M.V.D.); nikolaoumike@hotmail.com (M.N.); 2 Genekor Medical, Spaton 52, Ave., 15344 Athens, Greece; eirinipapad@GENEKOR.com (E.P.); gnasioulas@GENEKOR.com (G.N.); 3 Department of Chemistry, Aristotle University of Thessaloniki, University Campus, 54124 Thessaloniki, Greece; vouram@gmail.com (M.V.); sarli@chem.auth.gr (V.S.); 4 1st Oncology Department, “Saint Savas” Anticancer-Oncology Hospital, 11522 Athens, Greece; 5 Department of Electron Microscopy & Molecular Pathology, The Cyprus Institute of Neurology & Genetics, Nicosia 2371, Cyprus; mihalisp@cing.ac.cy; 6 The Cyprus School of Molecular Medicine, P.O. Box 23462, Nicosia 1683, Cyprus

**Keywords:** thiadiazole derivative, cancer immunogenic markers, MMR impairment, PD-L1 upregulation, tumor mutation burden

## Abstract

Microsatellite instability (MSI), tumor mutation burden (TMB), and programmed cell death ligand-1 (PD-L1) are particularly known as immunotherapy predictive biomarkers. MSI and TMB are closely related to DNA mismatch repair (MMR) pathway functionality, while the PD-L1 checkpoint mediates cancer cell evasion from immune surveillance via the PD-L1/PD-1 axis. Among all the novel triazolo[3,4-*b*]thiadiazole derivatives, the compound KA39 emerged as the most potent anticancer agent. In the present study, potential alterations in MSI, TMB, and/or PD-L1 expression upon cell treatment with KA39 are explored. We tested three MMR-deficient (DLD-1, LS174T, and DU-145) and two MMR-proficient (HT-29 and PC-3) human cancer cell lines. Our findings support KA39-induced PD-L1 overexpression in all cancer cell lines, although the most outstanding increase was observed in MMR-proficient HT-29 cells. MSI analysis showed that KA39 affects the MMR system, impairing its recognition or repair activity, particularly in MMR-deficient DLD-1 and DU-145 cells, enhancing oligonucleotide production. There were no remarkable alterations in the TMB between untreated and treated cells, indicating that KA39 does not belong to mutagenic agents. Taking together the significant in vitro anticancer activity with PD-L1 upregulation and MSI increase, KA39 should be investigated further for its implication in chemo-immunotherapy of cancer.

## 1. Introduction

Triazoles and thiadiazoles are heterocyclic compounds known for possessing a wide range of pharmacological properties, including anti-microbial, anti-inflammatory, anti-convulsant, antioxidant, radio-protective, anti-leishmanial, anti-viral, anti-hypertensive, anticancer, and anti-depressant activities [1,2,3]. A series of new chemical entities has been obtained by either modifying their heterocyclic rings at different positions or fusing their core structures together. The 1,2,4-triazolo[3,4-*b*][1,3,4]thiadiazoles and their derivatives, generated by the cyclization of 1,2,4-triazole and 1,3,4-thiadiazole on each other to form the fused system 1,2,4-triazolo[3,4-*b*][1,3,4]thiadiazoles derivatives, hold a broad spectrum of biological potentialities. Nonetheless, of particular interest remains the important anticancer/antitumor efficacy displayed by this class of heterocycles. The cytotoxic potency of 1,2,4-triazolo[3,4-*b*][1,3,4]thiadiazoles against several cancer cell lines has driven the synthesis of alternate compounds such as 3,6-disubstituted 1,2,4-triazolo[3,4-*b*][1,3,4]thiadiazoles [4]. Three newly synthesized triazolo[3,4-*b*]thiadiazole derivatives (TATDADs) induced efficient cell growth inhibitory activity against three human colorectal cancer cell lines [5]. Further, in vitro studies conducted on three human ovarian, two colorectal, and two prostate cancer cell lines have also demonstrated significant antiproliferative activity induced by these compounds [6]. Our studies support that topoisomerase IIα (topIIα) is a potential target of the tested TATDADs, which act as topIIα inhibitors on the phosphorylation at Ser-1106 that is closely associated with the decatenation activity of the enzyme. TATDADs induced the formation of supercoiled DNA by blocking topIIα’s either ATPase- or DNA-binding activity [5]. Of great interest is the inhibitory impact of these TATDADs on the phosphorylation of both AKT isoforms (AKT1 and AKT2), suggesting that TATDADs act as multitarget anticancer agents [6].

Even if the need for novel cytotoxic anticancer agents is constant, there are more revolutionary ways introduced to treat certain types of malignancies, such as cancer immunotherapy. Targeting immune checkpoints, including PD-L1/PD-1, induce more prolonged therapeutic responses than conventional chemotherapy, indicating that immunotherapy is a valuable tool in cancer cure [7]. Discriminating cancer patients with sensitivity to immune checkpoint blockade (ICB) therapies is necessary so as to ensure an antitumor response. Three biomarkers with promising predictivity have been established: programmed death-ligand 1 (PD-L1), microsatellite instability (MSI), and tumor mutational burden (TMB) [8,9].

**Figure 1:**
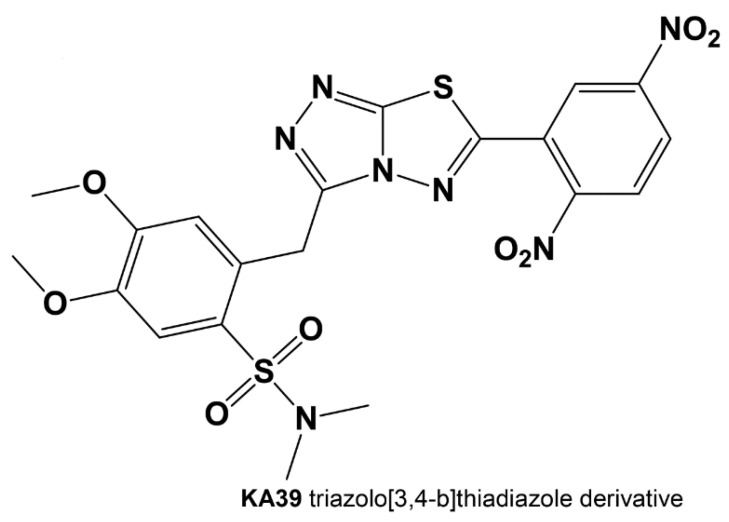
Chemical structure of the tested triazolo[3,4-*b*]thiadiazole derivatives (TATDAD), 2-((6-(2,5-dinitrophenyl)-[1,2,4]triazolo[3,4-b][1,3,4]thiadiazol-3-yl)methyl)-4,5-dimethoxy-*N*,*N*-dimethylbenzene sulfonamide (KA39).

The present study intends to explore potential alterations in PD-L1, MSI, and TMB predictive biomarkers upon cell treatment with KA39 (Figure 1; Table 1), a TATDAD with high anticancer activity. High MSI (MSI-H) has been observed in several cancers, including colorectal and prostate, with higher occurrence in colorectal cancer. In our study, three human colorectal and two prostate cancer cell lines were selected, of which some were mismatch repair deficient (dMMR)/MSI-H, while others were mismatch repair proficient (pMMR)/microsatellite stable (MSS) (Table 2). MSI assay was conducted in order to detect either qualitative or quantitative alterations in DNA fragments induced by KA39. Changes in PD-L1 expression were also evaluated in tumor cells treated with KA39, as well as the TMB, in order to investigate whether KA39 triggers an increase in the overall number of somatic mutations.

## 2. Materials and Methods

### 2.1. Cell Lines and Culture Conditions

Our study was conducted in five well-established human cancer cell lines: three colorectal adenocarcinoma (DLD-1, HT-29, and LS174T) and two prostate (PC-3 and DU-145) (Table 1). All cancer cell lines were obtained from the American Type Culture Collection (ATCC, Manassas, VA, USA) and cultivated in different culture media according to supplier instructions. All growth media were supplemented with 10% fetal bovine serum and 1% penicillin/streptomycin. All cancer cell lines were cultured as monolayers and maintained at 37 °C in a humidified 5% CO_2_ atmosphere.

### 2.2. Cell Treatment

Among several newly synthesized triazolo[3,4-*b*]thiadiazole derivatives that have been tested for anticancer activity, KA39 has been shown as the most potent, inducing significantly higher cytostatic and cytotoxic activity against all tested cancer cell lines. In addition to KA39, two triazolo[3,4-*b*]thiadiazoles derivatives with significant structural similarity (>95%), XK71 and XK57, were also tested. All molecules were synthesized according to procedures that we previously reported (Figure 1; Figure S21) [4,5].

### 2.3. In Vitro Antiproliferative Activity

The in vitro anticancer activity of KA39 against all cancer cell lines was evaluated using the 3-(4,5-dimethylthiazol-2-yl)-2,5-diphenyltetrazolium bromide quantitative colorimetric MTT assay, as previously reported [4,5]. Briefly, cells were seeded into a 96-well plate at a density of 8 × 10^3^ cells per well and maintained for 72 h. After 24 h of cell growth, cells were treated with KA39 in concentrations of 1–100 μM. Following 48 h of drug exposure, 50 μL of MTT (5 mg/mL) was added to each well and cells were incubated for 3 h. The absorbance of the converted dye was recorded at a wavelength of 540 nm on an ELISA reader (Versamax, Orleans, LA, USA).

MTT assay provides the required absorption values for determining three dose–response parameters, GI_50_, TGI, and IC_50_, using the linear progression method. All the experiments were carried out in triplicate. GI_50_ and TGI are drug concentrations that signify the cytostatic effect of a tested compound and induce 50% and 100% of cell growth inhibition, respectively; IC_50_ is the drug concentration that implies the cytotoxic effect of a tested compound and leads to a 50% decrease in cell viability [22,23]. The three parameters were determined using the mean of cell survival in all nine absorbance measurements, including control 24 h (Ct24), control 72 h (Ct72), and the seven drug concentrations (Tt72). The percentage of growth inhibition was calculated as [(Tt72x) − (Ct24)/(Ct72) − (Ct24)] × 100 for concentrations for which Tt72x > Ct24 and [(Tt72x) − (Ct24)/Ct24] × 100 for concentrations for which Tt72x < Ct24. GI_50_ was calculated as [(Tt72x) − (Ct24)/(Ct72) − (Ct24)] × 100 = 50; TGI as [(Tt72x) − (Ct24)/(Ct72) − (Ct24)] × 100 = 0; and IC_50_ as [(Tt72x) − (Ct24)/Ct24] × 100 = 50.

### 2.4. Flow Cytometric Analysis of Surface PD-L1 Expression

The impact of KA39 on PD-L1 expression was studied in four cancer cell lines, two colorectal (DLD-1 and HT-29) and two prostate (PC-3 and DU-145). Cells were seeded in a 6-well plate at a density of 5 × 10^5^ cells/well and maintained for 24 h at 37 °C in a humidified 5% CO_2_ atmosphere. After 24 h of cell growth, the culture medium was replaced with fresh medium; each cell line was treated with KA39 according to the defined values of the TGI concentration and IC_50_ (μΜ), while untreated cells served as controls (Figure 1; Table 1). Cells treated at the TGI concentration (μΜ) were allowed to grow for 72 h in contrast to IC_50_ (μΜ), in which cells were exposed for 48 and 72 h. Following drug treatment, cells were collected after being washed with ice-cold PBS (pH 7.4) (Sigma-Aldrich, St. Louis, MO, USA) and detached enzymatically with standard trypsinization. All centrifugations, performed at 1500 rpm for 5 min, included medium discard and washing steps with 2 mL of cold cell-staining buffer (BioLegend, San Diego, CA, USA). Subsequently, 25 × 10^4^ of cells were resuspended in 100 μL of cell-staining buffer and then stained with 5 μL of anti-PD-L1 antibody (PE/Cy7 anti-human CD274, Biolegend, San Diego, CA, USA). Cells were incubated at room temperature in the dark for 15 min and then resuspended with 800 μL οf cell-staining buffer. The expression of PD-L1 was analyzed on a flow cytometer (CyFlow^®^, SL, Partec, GmbH, Germany) using Partec Flomax software version 2.3 (Münster, Germany). For each sample, flow cytometric analysis was carried out in triplicate. The absolute values of the KA39-induced alterations in PD-L1 expression were reduced to a percentage, and controls (untreated cells) were defined as 100% of PD-L1 expression.

### 2.5. DNA Extraction

DNA extraction was carried out in frozen cell pellets using the QIAamp DNA Mini Kit (Qiagen, Hilden, Germany). According to manufacturer’s instructions, 200 μL of ATL buffer and 21 μL of proteinase K were added and then cell samples were incubated at 56 °C for 1 h. Following further incubation at 90 °C for 15 min, 200 μL of AL buffer as well as 200 μL of 100% ethanol were added. After brief vortex mixing, the DNA extract was transferred into a QIAamp Mini spin column (in a 2 mL collection tube) and centrifuged at 1200 rpm for 1 min. Subsequently, the QIAmp Mini spin column was placed into a new 2 mL collection tube and 500 μL of AW1 buffer was added. Once samples were centrifuged at 8000 rpm for 1 min, the QIAmp Mini spin column was placed into a new 2 mL collection tube and 500 μL of AW2 buffer was added. Like before, samples were centrifuged at 8000 rpm for 1 min. Afterward, the QIAamp Mini spin column was placed into a new 2 mL collection tube and centrifugation was carried out at full speed (14,000 rpm) for 3 min. The purified DNA was eluted by adding the appropriate volume of AE buffer (30–50 μL), and samples were finally centrifuged at 14,000 rpm for 1 min. The DNA concentration (ng/μL) of all samples was determined spectrophotometrically (NanoDrop2000, Thermo Fisher Scientific, Waltham, MA, USA).

### 2.6. MSI Fragment Analysis

Alterations in the MSI status were studied in three colorectal (DLD-1, HT-29, and LS174T) and two prostate (PC-3 and DU-145) human cancer cell lines upon treatment with KA39. Briefly, cells were seeded in a 6-well plate at a density of 3 × 10^5^ cells/well and cultured for 24 h at 37 °C in a humidified 5% CO_2_ atmosphere. After 24 h of cell growth, the culture medium was replaced by fresh medium and cells were subsequently treated with KA39 at the TGI concentration and IC_50_ (μΜ) for 48 h (Figure 1; Table 1). Following 48 h of drug exposure, the culture medium was discarded and cells were collected after being washed twice with ice-cold PBS (pH 7.4) (Sigma-Aldrich, St. Louis, MO, USA) and detached enzymatically with standard trypsinization. Afterward, cells were centrifuged twice at 2000 rpm for 5 min, while one further centrifugation was carried out in a microcentrifuge at 3000 rpm for 15 min. DNA was extracted and then subjected to multiplex PCR with fluorescently labeled primers obtained from Thermo Fisher Scientific (Waltham, MA, USA) [24] (Table 3). PCR was carried out using the commercial QIAGEN Multiplex PCR Kit (Qiagen, Hilden, Germany). Furthermore, MSI analysis was performed according to the NCI panel (Bethesda panel), which consists of two mononucleotide (BAT25 and BAT26) and three dinucleotide (D5S346, D17S250, and D2S123) repeats [25]. Following the manufacturer’s recommendations, each 20 μL of the PCR multiplex reaction mix was composed of the following: 12.5 μL of 2× Qiagen multiplex PCR master mix (HotStarTaq DNA Polymerase (5 units/µL), 6 mM MgCl_2_, pH (8.7), and dNTP mix (dATP, dCTP, dGTP, dTTP)), 2.5 μL of 10× MSI primer mix, template DNA whose volume was variable depending on the concentration of DNA isolated (ng/μL), and RNase-free water, which was added to reach a total volume of 20 μL. In the negative control, DNA was replaced by water. The PCR amplification program started with an activation step of HotStarTaq DNA Polymerase at 95 °C for 15 min, followed by 40 cycles of 30 s at 95 °C (denaturation), 90 s at 51 °C (annealing), and 60 s at 72 °C (extension), and a final extension step of 30 min at 60 °C (Veriti™ 96-Well Thermal Cycler; Thermo Fisher Scientific, Waltham, MA, USA). To ascertain whether the amplification of the expected amplicons was successful, PCR products were analyzed by capillary electrophoresis. More specifically, the PCR products, stained with ethidium bromide, were loaded on 2% agarose gel in 1× TAE buffer, run at 97 V for 45 min (electrophoresis system; Bio-Rad Laboratories, Hercules, CA, USA) and finally visualized using the MiniBis Pro (DNR Bio-Imaging Systems Ltd., Jerusalem, Israel). For fragment analysis, the PCR products were denatured by adding 14.5 μL of a mixture composed of GeneScan™ 500 LIZ™ Size Standard (Thermo Fisher Scientific, Waltham, MA, USA) and Hi-Di™ Formamide (Thermo Fisher Scientific, Waltham, MA, USA) to 1 μL of each diluted PCR product. To achieve DNA denaturation, samples were incubated at 95 °C for 3 min (Veriti™ 96-Well Thermal Cycler; Thermo Fisher Scientific, Waltham, MA, USA) and subsequently cooled down in the freezer for 3 min. DNA fragments were analyzed on the GeneScan 500 LIZ Genetic Analyzer (Thermo Fisher Scientific, Waltham, MA, USA), and data analysis was conducted using GeneMapper 4.0 software version 4.0 (Applied Biosystems, Foster City, CA, USA). MSI analysis was conducted by quantifying all DNA fragments per nanogram of DNA input in PCR.

### 2.7. Tumor Mutation Burden Assay

The TMB was determined in three colorectal cancer cell lines (DLD-1, HT-29, and LS174T) treated with KA39 at IC_50_ (μΜ) for 48 h (Figure 1; Table 1). The TMB was assessed by the Oncomine™ Tumor Mutation Load Assay (Thermo Fisher Scientific, Waltham, MA, USA), a targeted next-generation sequencing (NGS) assay that covers 1.65 Mb of genomic space, of which 1.2 Mb is exonic region and 0.45 Mb intronic. It analyzes 409 genes, providing accurate quantitation of somatic mutations used for TMB calculation in FFPE tissues (Table 4). From all samples, 19.2 ng of the DNA extracted was used as input for NGS library preparation (according to the manufacturer’s instructions, a minimum of 20 ng is required). Briefly, target regions were amplified using the 5× Ion AmpliSeq™ HiFi Mix (Thermo Fisher Scientific, Waltham, MA, USA) and Oncomine™ Tumor Mutation Load Assay (2×) manual library preparation primer pools 1 and 2 (Thermo Fisher Scientific, Waltham, MA, USA). Once target amplification reactions were completed, amplicons were digested with FUPA reagent and subsequently barcoded with the IonCode™ Barcode Adapters 1–384 Kit (Thermo Fischer Scientific, Waltham, MA, USA). The NGS libraries obtained were purified using Agencourt™ AMPure™ XP Reagent (Beckman Coulter, Life Sciences, Indianapolis, IN, USA) and quantified by qPCR using the Ion Library TaqMan® Quantitation Kit (Thermo Fisher Scientific, Waltham, MA, USA). Libraries were diluted to 50 pM before loading. Afterward, the libraries were combined by loading pools of 6 libraries on one Ion 550™ chip and sequenced on the Ion Gene Studio S5 Prime System (Thermo Fisher Scientific, Waltham, MA, USA). NGS data analysis was performed using Ion Reporter™ 5.10.1.0 software directly from within Torrent Suite™ 5.10.1 software (Thermo Fisher Scientific, Waltham, MA, USA), followed by manual inspection, along with the commercial analysis software Sequence Pilot version 4.3.0 (JSI medical systems, Ettenheim, Germany). The Ion Reporter pipeline, according to which the TMB was calculated, uses custom variant calling and germline variant filtering to accurately determine the number of exonic somatic mutations per megabase (Oncomine Tumor Mutation Load—w2.0—DNA—Single Sample).

### 2.8. Statistical Analysis

Student’s *t*-test was used to compare the level of significance between the experimental groups. Differences with a *p*-value less than 0.05 were considered statistically significant. Microsoft Excel version 2010 (Microsoft Hellas, Athens, Greece) was used.

## 3. Results

### MSI, PD-L1 Expression, and TMB Analysis in Human Colorectal and Prostate Cancer Cells upon Treatment with KA39

KA39 induced the most significant cytostatic and cytotoxic effects on the five tested human cancer cell lines (Table 1). However, XK71 and XK57 displayed low in vitro anticancer activity in the tested cancer cell lines, and no significant alterations in MSI and PD-L1 expression were impelled (*p* > 0.01) The alignment of cancer cell sensitivity to KA39 was DLD-1 > PC-3 > DU-145 > HT-29 > LS174T, with DLD-1 cells being more and LS174T less sensitive (*p* < 0.01). All results concerning the in vitro anticancer activity and changes in MSI and PD-L1 expression induced by XK71 and XK57 are provided as Supplementary Materials (Table S21–S23; Figures S22 and S23).

**Figure 2:**
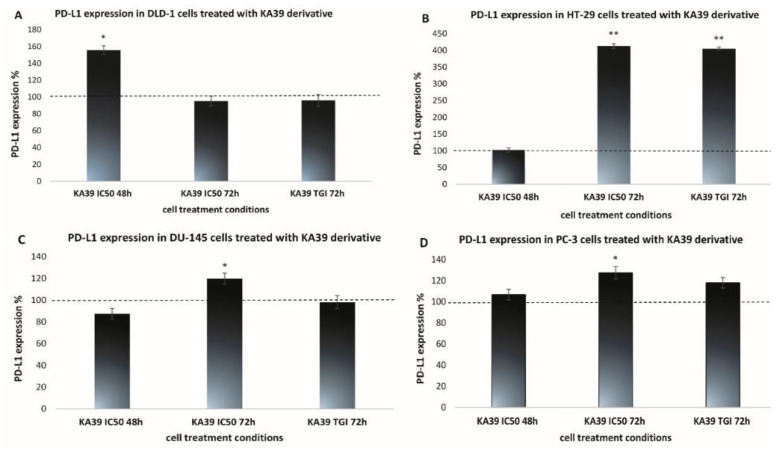
Percentage alterations in PD-L1 expression levels (mean ± SEM) induced by the KA39 triazolo[3,4-*b*]thiadiazole derivative. All four cancer cell lines were treated at IC_50_ (µM) for 48 and 72 h, as well as at the TGI concentration (μM) for 72 h. (**A**–**D**) illustrate the PD-L1 expression levels in DLD-1, HT-29, DU-145, and PC-3 cancer cells, respectively. The dotted line represents the control values defined as 100% of PD-L1 expression in each cancer cell line. Statistical significance level: * *p* < 0.05 and ** *p* < 0.01.

**Figure 3:**
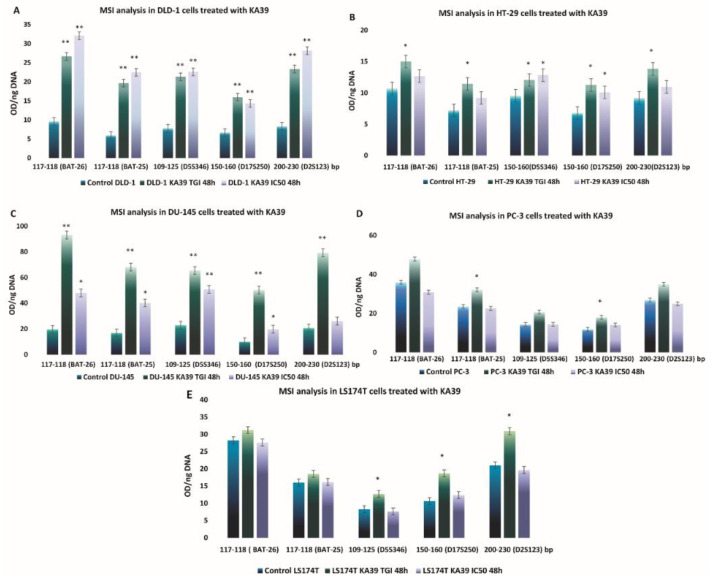
MSI alterations (mean ± SEM) induced by the KA39 triazolo[3,4-*b*]thiadiazole derivative (per ng DNA) in five human cancer cell lines. (**A**–**E**) demonstrate the MSI alterations in DLD-1, HT-29, DU-145, PC-3, and LS174T cancer cells, treated with KA39 at the TGI concentration and IC_50_ (μΜ) for 48 h, respectively. Quantitative MSI fragment analysis conducted in the Bethesda panel (BAT-26, BAT-25, D5S346, D17S250, and D2S123). Statistical significance level: * *p* < 0.05 and ** *p* < 0.01.

**Figure 4:**
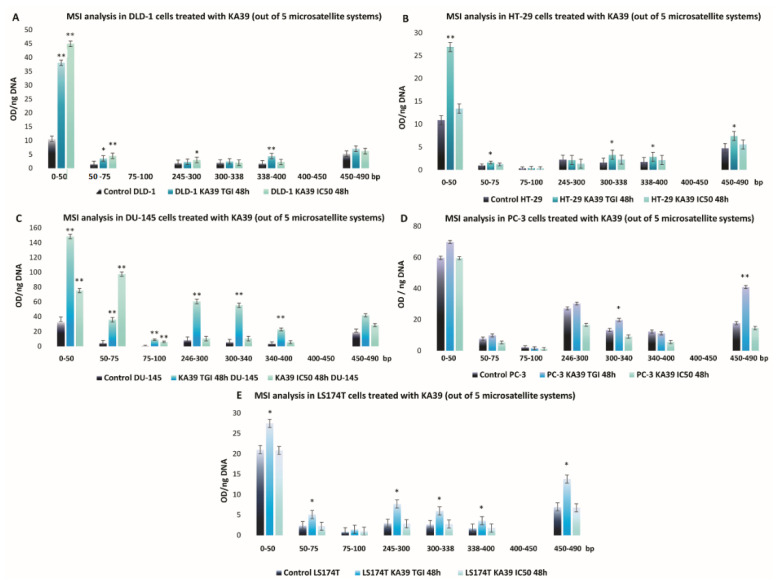
MSI alterations (mean ± SEM) induced by the KA39 triazolo[3,4-*b*]thiadiazole derivative (per ng DNA) in five human cancer cell lines. (**A**–**E**) represent the MSI alterations in DLD-1, HT-29, DU-145, PC-3, and LS174T cancer cells, treated with KA39 at the TGI concentration and IC_50_ (μΜ) for 48 h, respectively. Quantification of DNA fragments was carried out in eight additional regions (except the Bethesda panel), i.e., 0–50, 50–75, 75–100, 245–300, 300–338, 338–400, 400–450, 450–490 bp, which are considered by-products generated during GeneScan analysis. Statistical significance level: * *p* < 0.05 and ** *p* < 0.01.

On DLD-1 cells, PD-L1 expression levels significantly increased by 55.8% upon treatment with KA39 at a concentration of 9 μΜ (IC_50_) for 48 h (*p* < 0.05) (Figure 2A; Figures S1–S5; Tables S1–S5; Table 5). MSI analysis indicated that treatment with KA39, at both the TGI concentration (5 μΜ) and IC_50_ (9 μΜ) for 48 h, trigger a considerable increase in DNA fragments in the five major microsatellite markers, BAT-26, BAT-25, D5S346, D17S250, and D2S123 (*p* < 0.01) (Figure 3A). The increase in oligonucleotides was recorded as 3-fold higher in BAT-26, 4- to 5-fold higher in BAT-25, 2.5-fold higher in D5S346, and 3-fold higher in D17S250 and D2S123 microsatellites as compared to controls. Oligonucleotides also increased in the four additional regions by 4-fold in 0–50 bp (TGI and IC_50_; *p* < 0.01), from 3- to 5-fold in 50–75 bp (TGI and IC_50_; *p* < 0.05 and *p* < 0.01, respectively), 2-fold in 245–300 bp (IC_50_; *p* < 0.05), and 5-fold in 338–400 bp (TGI; *p* < 0.01) (Figure 4A). Regarding the TMB, no considerable alterations were demonstrated in the number of non-synonymous mutations when DLD-1 cells were treated with KA39 at IC_50_ (μM) for 48 h. However, a significant increase from 31.18 Muts/Mb in untreated to 80.93 Muts/Mb in treated cells was induced at the synonymous mutation number in DLD-1 cells (Table 6).

With respect to the HT-29 cancer cell line, treatment with KA39 increased PD-L1 expression by 313% and 304.9% when cells were treated at IC_50_ and the TGI concentration for 72 h, respectively (*p* < 0.01) (Figure 2B; Figures S6–S10; Tables S6–S10; Table 5). As MSI DNA fragment analysis suggests, a considerable increase in oligonucleotides was induced by KA39 in all five microsatellites (BAT-26, BAT-25, D5S346, D17S250, and D2S123). Cell treatment at the TGI concentration for 48 h led to augmentation of DNA fragments by 1.5-fold in BAT-26 (*p* < 0.05) and D2S123 (*p* < 0.05) and 2-fold in BAT-25 (*p* < 0.05). Exposure to both IC_50_ and the TGI concentration for 48 h increased oligonucleotides by 1.5- and 2-fold in D5S346 and D17S250 microsatellites, respectively (*p* < 0.05) (Figure 3B). Except the reference panel (Bethesda panel), DNA fragments were also elevated, upon treatment at the TGI concentration for 48 h, in the following seven regions: 0–50 bp (2.5-fold; *p* < 0.01), 50–75 bp (2-fold; *p* < 0.05), 300–338 bp (4-fold; *p* < 0.05), 338–400 bp (1.5-fold; *p* < 0.05), and 450–490 bp (2-fold; *p* < 0.05) (Figure 4B). Similar to DLD-1 cells, no alterations in the TMB was demonstrated after treatment of HT-29 cells with KA39 at IC_50_ for 48 h (Table 6).

The KA39-induced increment in PD-L1 expression was demonstrated in the DU-145 cancer cell line as well. Cell exposure to KA39 at IC_50_ for 72 h elevated PD-L1 expression levels by 19.86% (*p* < 0.05), whereas no considerable changes were observed under the remaining treatment conditions (*p* > 0.05) (Figure 2C; Figures S11–S15; Tables S11–S15; Table 5). Regarding MSI, treatment at both concentrations (TGI = 8 μΜ and IC_50_ =10.3 μΜ) for 48 h significantly increased oligonucleotides in all five microsatellites of the Bethesda panel as follows: 2 to 5 times in BAT-26 (TGI and IC_50_; *p* < 0.01 and *p* < 0.05, respectively), 2 to 3 in BAT-25 (TGI and IC_50_; *p* < 0.01 and *p* < 0.05, respectively), 2.5 to 3 in D5S346 (TGI and IC_50_; *p* < 0.01), 2 to 5 in D17S250 (TGI and IC_50_; *p* < 0.01 and *p* < 0.05, respectively), and 4 in D2S123 (TGI; *p* < 0.01) (Figure 3C). In addition to the Bethesda panel, oligonucleotides increased 40-fold in 0–50 bp (TGI and IC_50_; *p* < 0.01), 20-fold in 50–75 bp (TGI and IC_50_; *p* < 0.01), 10-fold in 75–100 bp (TGI and IC_50_; *p* < 0.01), 6-fold in 246–300 bp (TGI; *p* < 0.01), 5-fold in 300–340 bp (TGI; *p* < 0.01), and 4-fold in 340–400 bp (TGI; *p* < 0.01) (Figure 4C).

In the PC-3 cancer cell line, treatment with KA39 at IC_50_ for 72 h resulted in a significant increase in PD-L1 expression (27.28%; *p* < 0.05) (Figure 2D; Figures S16–S20; Tables S16–S20; Table 5). As demonstrated in Figure 3D and Figure 4D, a considerable increase in DNA fragments was impelled when PC-3 cells were treated with KA39 at the TGI concentration for 48 h, elevating oligonucleotides by 1.5-fold in BAT-25 (*p* < 0.05) and D17S250 (*p* < 0.05) microsatellites, as well as in the regions of 300–340 bp (*p* < 0.05) and 450–490 bp (*p* < 0.01) in which DNA fragments increased by 1.5- and 2-fold, respectively.

MSI and TMB alterations induced by KA39 were also studied in the LS174T human cancer cell line. Treatment at the TGI concentration for 48 h increased DNA fragments by 1.5-fold in D5S346 (*p* < 0.05), 2-fold in D17S250 (*p* < 0.05), and 1.5-fold in D2S123 (*p* < 0.05) microsatellites (Figure 3E). Beyond the Bethesda panel, increased oligonucleotides, as compared with untreated cells, were detected in the regions of 0–50 bp (1.5-fold higher; *p* < 0.05), 50–75 bp (2.5-fold higher; *p* < 0.05), 245–300 bp (2-fold higher; *p* < 0.05), 300–338 bp (2-fold higher; *p* < 0.05), and 338–400 bp (2-fold higher; *p* < 0.05) upon exposure to KA39 under the same treatment condition (Figure 4E). With reference to the TMB, no significant alterations were recorded upon treatment with KA39 at IC_50_ for 48 h, according to the TMB values (Table 6).

Among all human cancer cell lines included in our experimental studies, DLD-1, DU-145, and PC-3 cells were the most sensitive to KA39, as TGI and IC_50_ values indicate (Table 1). Nevertheless, HT-29 cancer cells, the cell line less susceptible to KA39, exhibited the most outstanding increase in PD-L1 expression levels in comparison with DLD-1, DU-145, and PC-3 cells. However, MSI appeared to be significantly enhanced in DLD-1 and DU-145 cancer cells in which KA39 generated a higher cytostatic and cytotoxic effect. Comparing with DLD-1 and DU-145 cells, less meaningful but statistically significant MSI enhancement was detected in HT-29, LS174T, and PC-3 cells. With reference to TMB assay and non-synonymous mutations, no considerable alterations were displayed by the tested cancer cell lines, as shown by the TMB values of untreated and treated cancer cells.

## 4. Discussion

PD-L1, also termed as CD274 or B7-H1, is the physiological ligand of the PD-1 receptor, and both are most important immune checkpoints. PD-L1 expressed by tumor cells is bound to PD-1, which is located on activated T cells. Cancer cells, through PD-L1/PD-1 interaction, are capable of suppressing the tumor-reactive T cells and evading cancer immune surveillance [26,27]. Even though chemotherapy provides important therapeutic benefits, harmful effects may be induced on anticancer immunity, such as the drug-induced PD-L1 expression in cancer cells. To the best of our knowledge, current studies are addressing the effects of anticancer agents on PD-L1 expression in a variety of cancers, including pancreatic, ovarian, and breast [27,28]. Moreover, there appears to be a dose-dependent relationship between PD-L1 upregulation and chemopreventive or cytotoxic agents as, for example, in decitabine and cisplatin administered in leukemia and hepatoma cells, respectively [28,29]. Qin et al. [29] showed that PD-L1 is overexpressed in H22 hepatoma cells following exposure to cisplatin at a concentration less than IC_50_. According to our findings, all four cancer cell lines treated with KA39 showed significantly elevated PD-L1 expression levels, though the most impressive increase was induced in HT-29 cancer cells (Figure 2A–D). HT-29 cells were less sensitive to KA39 with the TGI concentration and IC_50_ being approximately 2-fold higher compared to those of DLD-1, DU-145, and PC-3 cells (Table 1). PD-L1 overexpression in the treated HT-29 cells occurred in a dose-dependent manner.

It is suggested that oncogenic signaling pathways such as RAS-RAF-MEK-ERK, PI3K-AKT-mTOR, JAK-STAT, and NF-κB are strongly involved in the anticancer agent-mediated PD-L1 expression probably by a signaling crosstalk. In view of the PI3K-AKT-mTOR pathway, inhibition of either PI3K or its downstream signaling molecule AKT leads to repression of PD-L1 expression in tumor cells [30,31,32]. On the contrary, as shown in experimental studies using breast cancer cell lines, loss of phosphatase and tensin homolog (PTEN), due to mutation or depletion, leads to PI3K activation, which, in turn, upregulates the expression of PD-L1 [31,32]. Regarding the PTEN-deficient PC-3 cell line [21], KA39-induced PD-L1 expression was barely higher than in PTEN wild-type DU-145 cells; however, it was not as notable as in HT-29 and DLD-1 cells (both PTEN wild type), in which PD-L1 was upregulated to a far greater extent (Figure 2A,B). Moreover, taking into consideration that the KA39 derivative behaves as an AKT inhibitor [6], downregulation of PD-L1 would be expected. Nevertheless, our results support an acceleration of PD-L1 expression in response to KA39 exposure irrespective of the PTEN status and AKT inhibition, signifying a distinct mechanism of action.

Accumulating evidence suggests that PD-L1 upregulation may occur in the context of DNA-damage-induced signaling in tumor cells [33]. As we have previously reported, KA39 induces topIIα inhibition as well [5]. TopIIα inhibitors, as DNA-damaging agents, stimulate the expression of type I IFNs and other cytokines via the activation of the cGAS-STING-TBK1-IRF3 pathway [34]. Furthermore, an association of the cGAS-STING pathway with PD-L1 upregulation has been reported [35]. Wang et al. [34] also found an immune response impelled by the topoisomerase II inhibitor, in particular teniposide-induced PD-L1 expression in multiple cancer cells in vitro. Mechanically, the cytoplasmic DNA, caused by exposure to topII inhibitors, serves as a DNA damage signal, which can be sensed by cyclic GMP-AMP synthase (cGAS). cGAS binding to cytosolic DNA promotes the synthesis of the second messenger cyclic GMP-AMP (cGAMP), which binds to the adaptor protein Stimulator of IFN Gene (STING). The activated STING recruits a signaling cascade that triggers the transcription of type I IFNs genes [36]. It is thought that type I INFs regulate PD-L1 expression through the JAK1/JAK2-STAT1/STAT2/STAT3-IRF1 axis, with IRF1 being bound to the PD-L1 promoter and hence triggering its production [33,34,37].

The MSI phenomenon emerges from a deficient mismatch repair (dMMR) mechanism, which is responsible for the accumulation of mutations in the genome’s short tandemly repeats (STRs), termed as microsatellites [9]. There are three distinct types of MSI phenotypes linked to MMR functionality: (1) MSI-H associated with a dMMR system, (2) low microsatellite instability (MSI-L), and (3) MSS, with the last two being related to pMMR status [38]. The MSI-H phenotype appears in several sporadic cancers, including colorectal, gastric, small intestine, urothelial, endometrial, and, more rarely, prostate [9,39]. Loss of MMR function contributes to enhanced chemoresistance as dMMR cells are less sensitive to commonly used chemotherapeutic drugs such as alkylating agents, platinum compounds, topoisomerase poisons, and purine analogues [40]. However, anti-PD-1 and anti-PD-L1 immunotherapies are much more efficient in dMMR/MSI-H tumors. Concurrent coexistence of neoantigens produced by MMR mutations, with PD-L1 immune checkpoint expression, provides a breeding ground for MSI-H tumors to be treated with anti-PD-L1 therapy [38,41,42,43].

The MSI condition is linked to an impaired MMR mechanism whose dysfunctionality results from mutations, either germline or spontaneous, in MMR genes [12]. The MMR pathway, responsible for the maintenance of genomic integrity, undertakes the repair of postreplicative DNA base mismatches as well as inserted/deleted loops incorporated into microsatellites. Four key genes support MMR’s functionality: mutL homologue 1 (MLH1), postmeiotic segregation increased 2 (PMS2), mutS homologue 2 (MSH2), and mutS6 (MSH6) [44]. The MMR mechanism is initiated with the mismatch being recognized by the MutSα heterodimer (MSH2/MSH6). Afterward, MutSα interacts with the MutLα complex (MLH1/PMS2), forming a “sliding clamp” that moves up and down on the DNA sequence that contains the mismatch. This sliding clamp acts in an ATP-hydrolysis-dependent manner [45]. Once the daughter strand has been identified, the MutSα/MutLa complex guides DNA Exonuclease 1 (EXO1) in order to carry out an excision at the mismatch site. The repair process is completed once the removed DNA sequence is replaced by DNA polymerase δ and the remaining gaps are sealed by DNA ligase I [46,47]. Microsatellites are known for their extreme vulnerability to errors that arise from DNA replication, recombination, or external sources such as radiation or chemical agents [44].

Our study points out a considerable accumulation of oligonucleotides in all five cancer cell lines treated with the anticancer agent KA39, comprising dMMR as well as pMMR cancer cells. Nonetheless, augmentation of DNA fragments, impelled by KA39, was remarkably higher in dMMR DLD-1 and DU-145 cells than in pMMR HT-29 and PC-3 cells, even in dMMR LS174T cells. Furthermore, it is noteworthy that MSI was enhanced in terms of quantity, as more oligonucleotides resulted from KA39 exposure, whereas no alterations were revealed in the relocation of DNA fragments. Each MSI-H cancer cell line carries specific MMR deficiencies (Table 2). DLD-1 cells are dMMR cancer cells bearing a missense mutation in the MSH6 gene, which is responsible for complete loss of the protein. In the absence of the MSH6 protein, single-base mismatches cannot be repaired, though the MMR pathway retains its functionality in general terms. The lack of MSH6 expression does not influence the other MutS proteins, as MSH2 counterbalances its loss by binding to MSH3 [48]. Thus, it can be suggested that in DLD-1 cells, the MMR pathway maintains its recognition activity to a certain extent. With respect to dMMR DU-145 cells, the MutLα heterodimer is completely absent, as both MLH1 and PMS2 proteins are lacking, and consequently the MMR repair activity is significantly diminished. It is interesting to note that the loss of the MLH1 protein has a crucial impact on the MMR mechanism as it leads to inactivation of MutLα’s endonuclease activity, degradation of PMS2, and, hence, an extreme accumulation of errors [17,18,49]. With regard to dMMR LS174T cells, all proteins (MSH2, MSH6, PMS2, and MLH1) are expressed at low levels. However, studies have shown that MLH1 appears to a far lesser extent, probably indicating an attenuated activity of the MutLa complex [50]. Presumably, the KA39 agent further deteriorated the MMR mechanism, mainly in dMMR cells, by disrupting the recognition or repair activity of the MMR mechanism. Nevertheless, the exact molecular mechanism through which KA39 disrupts the MMR pathway is unclear. MSI-H cancer cell lines such as DLD-1 (MSH6 deficiency) and DU-145 (MLH1 and PMS2 deficiencies) cells showed a greater susceptibility to KA39 than the MSS cancer cell line HT-29, pointing out a kind of synthetic lethality. However, the genetic and molecular profile of a cell line influence its cellular response to an anticancer drug; MSS PC-3 and MSI-H LS174T cells share similar sensitivity to KA39 as MSI-H DU-145 and MSS HT-29 cells, respectively (Table 1).

Studies related to the MMR pathway support an association of MMR deficiency with resistance to topII inhibitors, though the findings around this area are controversial [51,52]. In pMMR cancer cells, the cleavage complex, induced by a topII inhibitor, is recognized by the MMR pathway, activating eventually the apoptotic procedure. In contrast, the genomic instability of dMMR cells increases the mutation rates in the topII gene, leading, as a consequence, to mutated topII unable to be bound to a topII inhibitor [53,54]. As previously discussed, our results regarding the drug sensitivity to KA39 exhibit heterogeneity, which may be attributable to their specific characteristics.

A growing body of evidence supports the implication of AKT in the MMR pathway via PMS2, a component of the MutLα heterodimer. More particularly, binding of the phosphorylated AKT Ser473 to PMS2 leads to the degradation of MutLα’s component, inducing genomic instability and DNA damage. By contrast, inhibition of AKT ensures the stability and nuclear localization of PMS2. It appears that cells with hyperactivated AKT carry ineffective MMR responsible for enhanced accumulation of mutations, drug resistance, and inactivation of the apoptotic procedure [55,56]. Even though KA39 acts as a potent AKT inhibitor as well [6], the MMR pathway was significantly impaired, pointing out that KA39 is not a pure AKT inhibitor. Probably, KA39 targets other elements of the repair mechanism.

The TMB represents the number of exonic non-synonymous mutations per megabase (Mb) [57]. Given that the TMB serves as a measure of somatic coding mutations, the volume of neoantigens can be evaluated and, by extension, the responsiveness to ICB therapy can be predicted. It is interesting to note that a higher number of mutations signifies higher response rates, as shown in MSI-H tumors known for their immunogenicity, which is associated with an increasing TMB [42,58]. KA39 produced no direct alterations on the TMB and on the non-synonymous mutation number in the tested cancer cell lines independent of their MMR status. However, a significant increase (>2-fold) of unknown importance was induced at the synonymous mutation number in DLD-1 cells, which bear an MSH6 deficiency (Table 6).

## 5. Conclusions

The impact of the KA39 triazolo[3,4-*b*]thiadiazole derivative was investigated on three predictive biomarkers for cancer immunotherapy: PD-L1, MSI, and TMB. According to our findings, a notable increase in PD-L1 expression and MSI was demonstrated, presumably in the context of DNA damage introduced by KA39, while no changes in the TMB were induced. Conclusively, KA39 cannot be considered a mutagenic agent, and microsatellite errors were accumulated by destabilizing the MMR mechanism during cancer cell treatment. Altogether, the significant in vitro anticancer activity in combination with PD-L1 upregulation and MSI enhancement implies that KA39 is a promising anticancer agent able to be developed for cancer chemo-immunotherapy.

## Figures and Tables

**Table 1:** GI_50_, TGI, and IC_50_ values of KA39, defined according to MTT assay, in human prostate and colorectal cancer cell lines.

Cancer Cell Lines	GI_50_ (μΜ)	TGI (μΜ)	IC_50_ (μΜ)
DLD-1	3 ± 0.52	5 ± 0.76	9 ± 0.76
HT-29	11.5 ± 0.8	15.9 ± 0.55	19.5 ± 0.9
LS174T	12 ± 1.52	16.5 ± 1.25	21.5 ± 1.5
PC-3	5 ± 0.15	8.4 ± 0.1	12 ± 0.1
DU-145	5.8 ± 0.2	8 ± 0.4	10.3 ± 1.8

**Table 2:** Description of histotypes and MMR protein expression of the five human cancer cell lines included in our study.

Cancer Type	Human Cell Line Designation	MSI Status	MMR Deficiency	PTEN	References
Colorectal adenocarcinoma, Dukes’ type C	DLD-1	MSI-H	MSH6 deficiency	Wild type	[10,11,12]
Colorectal adenocarcinoma	HT-29	MSS	-	Wild type	[10,11,13]
Colorectal adenocarcinoma, Dukes’ type B	LS174T	MSI-H	MLH1 deficiency	Wild type	[10,11,14,15]
Prostate carcinoma	DU-145	MSI-H	PMS2 and MLH1 deficiency	Wild type	[14,16,17,18,19]
Prostate adenocarcinoma, grade IV	PC-3	MSS	-	PTEN deficiency (homologous deletion)	[16,20,21]

**Table 3:** Primers and characteristics of microsatellite loci.

Repeat Type	Chromosomal Location	Repeat Motif	Primer Sequence (5′→3′)	Size (bp)
**Mononucleotide**				
BAT25	4q12	TTTT.T.TTTT.(T)_7_.A(T)_25_	TCGCCTCCAAGAATGTAAGT	~90
			TCTGCATTTTAACTATGGCTC	
BAT26	2p	(T)_5_…..(A)_26_	TGACTACTTTTGACTTCAGCC	~80–100
			AACCATTCAACATTTTTAACCC	
**Dinucleotide (non-complex)**				
D5S346 (APC)	5q21/22	(CA)_26_	ACTCACTCTAGTGATAAATCG	96–122
			AGCAGATAAGACAGTATTACTAGTT	
**Dinucleotide (complex)**				
D17S250 (Mfd15CA)	17q11.2-q12	(TA)_7_………………(CA)_24_	GGAAGAATCAAATAGACAAT	~150
			GCTGGCCATATATATATTTAAACC	
D2S123 (AFM093xh3)	2p16	(CA)_13_TA(CA)_15_(T/GA)_7_	AAACAGGATGCCTGCCTTTA	197–227
			GGACTTTCCACCTATGGGAC	

**Table 4:** List of all genes contained in the Oncomine Tumor Mutation Load assay.

ABL2	CD79A	EPHB1	GRM8	LIFR	MYH9	PMS1	SOX2	WAS	GNAS	ATRX	TSC2
ACVR2A	CD79B	EPHB4	GUCY1A2	LPHN3	NCOA1	POT1	SSX1	WHSC1	HFN1A	BAP1	WT1
ADAMTS20	CDC73	EPHB6	HCAR1	LPP	NCOA2	POU5F1	STK36	WRN	HRAS	CDK12	
AFF1	CDH1	ERCC1	HIF1A	LRP1B	NCOA4	PPARG	SUFU	XPA	IDH1	CDKN2A	
AFF3	CDH11	ERCC3	HLF	LTF	NFKB1	PPP2R1A	SYK	XPC	IDH2	CDKN2B	
AKAP9	CDH2	ERCC4	HOOK3	LTK	NFKB2	PRDM1	SYNE1	XPO1	JAK2	CEBPA	
APC	CDH20	ERCC5	HSP90AA1	MAF	NIN	PRKAR1A	TAF1	XRCC2	KOR	CHEK1	
ARID2	CDH5	ERG	HSP90AB1	MAFB	NKX2-1	PRKDC	TAF1L	ZNF384	KIT	CHEK2	
ARNT	CDK8	ETS1	ICK	MAGEA1	NLRP1	PSIP1	TAL1	ZNF521	KRAS	CREBBP	
ATF1	CDKN2C	ETV1	IGF1R	MAGl1	NOTCH4	PTGS2	TBX22	ABL1	MAP2K1	DNMT3A	
AURKA	CIC	ETV4	IGF2	MALT1	NSD1	PTPRD	TCF12	AKT1	MAP2K2	FANCA	
AURKB	CKS1B	EXT1	IGF2R	MAML2	NUMA1	PTPRT	TCF3	AKT2	MAP2K4	FANCD2	
AURKC	CMPK1	EXT2	IKBKB	MAP3K7	NUP214	RALGDS	TCF7L1	AKT3	MAPK1	FBXW7	
BAI3 COL	COL1A1	FAM123B	IKBKE	MAPK8	NUP98	RARA	TCF7L2	ALK	MET	MLH1	
BCL10	CRBN	FANCC	IKZF1	MARK1	PAK3	RECQL4	TCL1A	AR	MPL	MSH2	
BCL11A	CREB1	FANCF	IL2	MARK4	PARP1	REL	TET1	AXL	MTOR	MSH6	
BCL11B	CRKL	FANCG	IL21R	MBD1	PAX3	RHOH	TFE3	BRAF	MYC	NBN	
BCL2	CRTC1	FANCJ	IL6ST	MCL1	PAX5	RNASEL	TGFBR2	CBL	MYCN	NF1	
BCL2L1	CSMD3	FAS	IL7R	MDM2	PAX7	RNF2	TGM7	CCND1	NFE2L2	NF2	
BCL2L2	CTNNA1	FH	ING4	MDM4	PAX8	RNF213	THBS1	CDK4	NRAS	NOTCH1	
BCL3	CTNNB1	FLCN	IRF4	MEN1	PBRM1	RPS6KA2	TIMP3	CDK6	NTRK1	NOTCH2	
BCL6	CYLD	FLl1	IRS2	MITF	PBX1	RRM1	TLR4	CSF1R	NTRK3	NPM1	
BCL9	CYP2C19	FLT1	ITGA10	MLL	PDE4DIP	RUNX1T1	TLX1	DDR2	PDGFRA	PALB2	
BCR	CYP2D6	FLT4	ITGA9	MLL2	PDGFB	SAMD9	TNFAIP3	EGFR	PDGFRB	PIK3R1	
BIRC2	DAXX	FN1	ITGB2	MLL3	PER1	SBDS	TNFRSF14	ERBB2	PIK3CA	PMS2	
BIRC3	DCC	FOXL2	ITGB3	MLLT10	PGAP3	SDHA	TNK2	ERBB3	PIK3CB	PTCH1	
BIRC5	DDB2	FOXO1	JAK1	MMP2	PHOX2B	SDHB	TOP1	ERBB4	PTPN11	PTEN	
BLM	DDIT3	FOXO3	JAK3	MN1	PIK3C2B	SDHC	TPR	ERCC2	RAF1	RADSO	
BLNK	DEK	FOXP1	JUN	MRE11A	PIK3CD	SOHD	TRIM24	ESR1	RET	RB1	
BMPR1A	DICER1	FOXP4	KAT6A	MTR	PIK3CG	SEPT9	TRIM33	EZH2	ROS1	RUNX1	
BRD3	DPYD	FZR1	KAT6B	MTRR	PIK3R2	SGK1	TRIP11	FGFR1	SF3B1	SETD2	
BTK	DST	G6PD	KDM5C	MUC1	PIM1	SH2D1A	TRRAP	FGFR2	SMO	SMARCA4	
BUB1B	EML4	GATA1	KDM6A	MUTYH	PKHD1	SMAD2	TSHR	FGFR3	SRC	SMARCB1	
CARD11	EP300	GATA2	KEAP1	MYB	PLAG1	SMAD4	UBR5	FGFR4	ARID1A	STK11	
CASC5	EP400	GATA3	KLF6	MYCL1	PLCG1	SMUG1	UGT1A1	FLT3	ASXL1	TET2	
CCND2	EPHA3	GDNF	LAMP1	MYD88	PLEKHG5	SOCS1	USP9X	GNA11	ATM	TP53	
CCNE1	EPHA7	GPR124	LCK	MYH11	PML	SOX11	VHL	GNAQ	ATR	TSC1	

**Table 5:** The absolute values of PD-L1 expression as defined by flow cytometric analysis in untreated (control) and treated cells with the KA39 derivative at IC_50_ (μΜ) for 48 and 72 h, as well as at the TGI concentration (μΜ) for 72 h. The units of measurements are expressed as the mean of immunofluorescence intensities counted per cell.

Cancer Cell Lines	PD-L1 Expression				
	Control 48 h	KA39 IC_50_ 48 h	KA39 IC_50_ 72 h	Control 72 h	KA39 TGI 72 h
DLD-1	12.92 ± 0.64	20.14 ± 1.0	12.3 ± 0.61	23.19 ± 1.39	22.27 ± 1.11
HT-29	19.56 ± 1.36	20.13 ± 1.61	80.82 ± 4.04	19.4 ± 1.35	78.56 ± 5.49
DU-145	27.99 ± 1.67	24.48 ± 2.2	33.55 ± 2.34	23.46 ± 1.17	23.02 ± 1.61
PC-3	25.91 ± 1.81	27.66 ± 2.7	32.98 ± 3.2	23.93 ± 1.91	28.21 ± 2.53

**Table 6:** TMB values in untreated (control) and treated colorectal cancer cells with the KA39 derivative at IC_50_ (μΜ) for 48 h.

Cancer Cell Lines	Tumor Mutation Burden (TMB)			
	Control 48 h		KA39 IC_50_ (μΜ) 48 h	
	Non-Synonymous Mutations	Synonymous Mutations	Non-Synonymous Mutations	Synonymous Mutations
DLD-1	204.17 Muts/Mb	31.18 Muts/Mb	198.28 Muts/Mb	80.93 Muts/Mb
HT-29	10.05 Muts/Mb	1.67 Muts/Mb	10.01 Muts/Mb	1.67 Muts/Mb
LS174T	67.48 Muts/Mb	3.48 Muts/Mb	68.06 Muts/Mb	3.62 Muts/Mb

## Data Availability

Data are available upon request.

## Supplementary Materials

The following are available online at https://www.mdpi.com/article/10.3390/pharmaceutics13060885/s1, Figures S1–S20: Flow cytometric analysis data, Figure S21: Chemical structures of XK57 and XK71, Figure S22: MSI alterations induced by XK57 derivative in five human cancer cell lines, Figure S23: MSI alterations induced by XK71 derivative in five human cancer cell lines, Tables S1–S20: Flow cytometric analysis data—Regions analysis, Table S21: Cytostatic and cytotoxic effects induced by XK57 and XK71 on the tested human cancer cell lines, Table S22: The absolute values of PD-L1 expression as defined by flow cytometric analysis in untreated and treated cells with XK57 derivative, Table S23: The absolute values of PD-L1 expression as defined by flow cytometric analysis in untreated and treated cells with XK71 derivative.
