# Supplementary material for: Effects of a Novel Thiadiazole Derivative with High Anticancer Activity on Cancer Cell Immunogenic Markers: Mismatch Repair System, PD-L1 Expression, and Tumor Mutation Burden
Source: Pharmaceutics. 2021 Jun 15;13(6):885. doi: 10.3390/pharmaceutics13060885 (PMC8232699; doi:10.3390/pharmaceutics13060885)
Supplement: Supplementary file 1 [file pharmaceutics-13-00885-s001.zip › pharmaceutics-1243911-supplementary.pdf]

pharmaceutics

MDPI

Supplementary Materials: Effects of a Novel Thiadiazole Derivative with High Anticancer Activity on Cancer Cell Immunogenic Markers: Mismatch Repair System, PD-L1 Expression, and Tumor Mutation Burden

Sofia Sagredou, Panagiotis Dalezis, Eirini Papadopoulou, Maria Voura, Maria V. Deligiorgi, Michail Nikolaou, Mihalis I. Panayiotidis, George Nasioulas, Vasiliki Sarli and Dimitrios T. Trafalis

Flow Cytometric Analysis Data

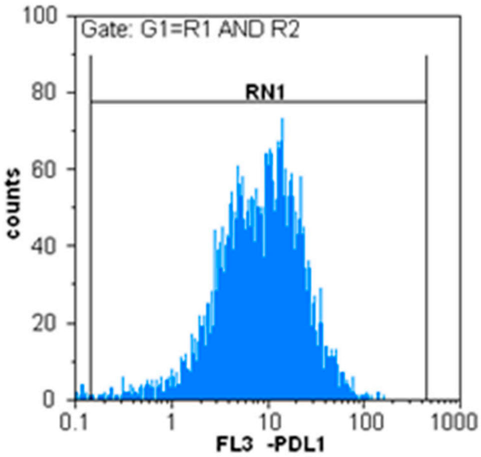

Figure S1. Flow cytometric analysis of PD-L1 expression on untreated DLD-1 cancer cells cultured for 48 h.

Table S1. Region analysis – calculations as resulted from the CyFlow® Partec Report software.

Region	Gate	Ungated	Count	Count/mL	%Gated	Mean-x	CV-x%	Mean-y	CV-y%
R1	<None>	6730	6730	-	65.61	2.37	65.80	12.13	47.06
RN1	G1	9784	3989	-	97.34	**12.92**	104.62	-	-
R2	<None>	4283	4283	-	41.75	0.52	488.28	0.26	10.38

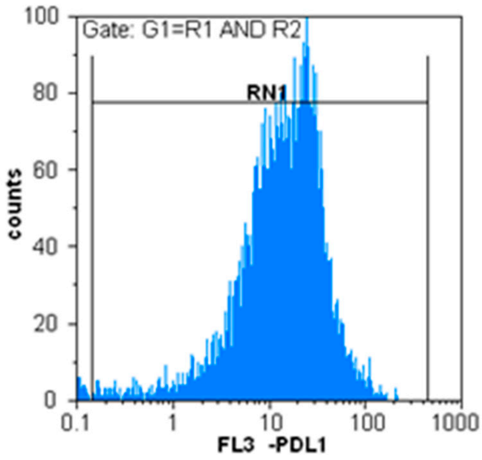

Figure S2. Flow cytometric analysis of PD-L1 expression on DLD-1 cancer cells treated with KA39 at IC_{50} concentration (μM) for 48 h.

Table S2. Region analysis – calculations as resulted from the CyFlow® Partec Report software.

Region	Gate	Ungated	Count	Count/mL	%Gated	Mean-x	CV-x%	Mean-y	CV-y%
R1	<None>	7320	7320	-	62.06	2.99	68.31	16.15	40.74
RN1	G1	11050	4991	-	93.69	**20.14**	97.27	-	-
R2	<None>	5472	5472	-	46.39	0.67	396.09	0.26	9.78

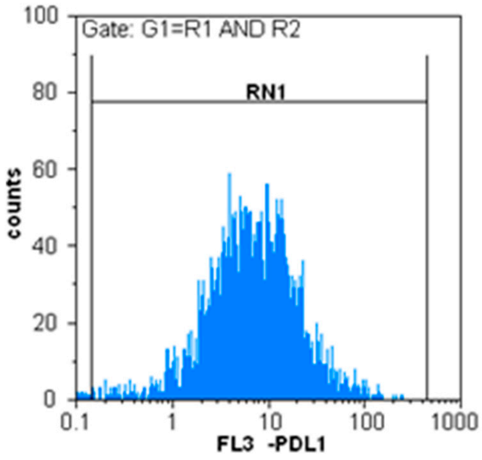

Figure S3. Flow cytometric analysis of PD-L1 expression on DLD-1 cancer cells treated with KA39 at IC_{50} concentration (μM) for 72 h.

Table S3. Region analysis – calculations as resulted from the CyFlow® Partec Report software.

Region	Gate	Ungated	Count	Count/mL	%Gated	Mean-x	CV-x%	Mean-y	CV-y%
R1	<None>	6798	6798	-	66.50	2.04	71.35	10.39	44.68
RN1	G1	9803	3383	-	97.05	**12.30**	144.61	-	-
R2	<None>	3628	3628	-	35.49	0.35	583.91	0.26	9.72

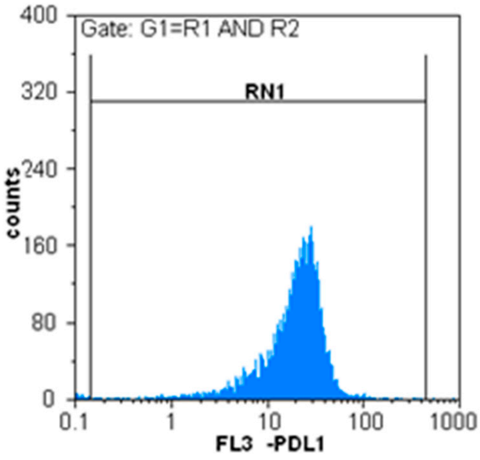

Figure S4. Flow cytometric analysis of PD-L1 expression on untreated DLD-1 cancer cells cultured for 72 h.

Table S4. Region analysis – calculations as resulted from the CyFlow® Partec Report software.

Region	Gate	Ungated	Count	Count/mL	%Gated	Mean-x	CV-x%	Mean-y	CV-y%
R1	<None>	11511	11511	-	55.75	3.21	64.50	18.92	42.29
RN1	G1	19656	5599	-	97.14	**23.19**	75.96	-	-
R2	<None>	6010	6010	-	29.11	2.14	267.75	0.28	10.09

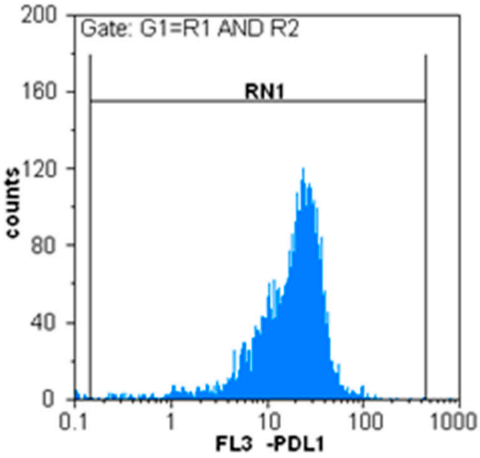

Figure S5. Flow cytometric analysis of PD-L1 expression on DLD-1 cancer cells treated with KA39 at TGI concentration (μM) for 72 h.

Table S5. Region analysis – calculations as resulted from the CyFlow® Partec Report software.

Region	Gate	Ungated	Count	Count/mL	%Gated	Mean-x	CV-x%	Mean-y	CV-y%
R1	<None>	8954	8954	-	44.08	2.85	56.68	17.68	39.68
RN1	G1	19308	4172	-	97.20	**22.27**	77.37	-	-
R2	<None>	4711	4711	-	23.19	1.39	328.99	0.28	9.77

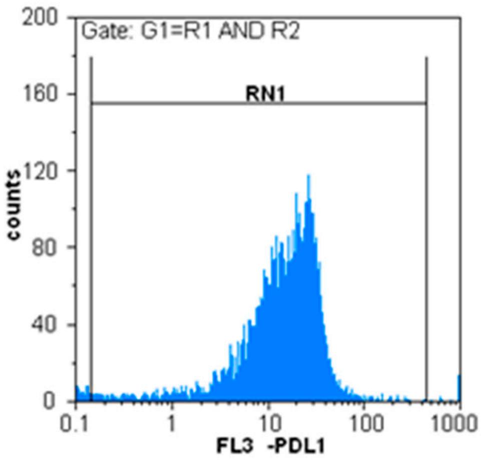

Figure S6. Flow cytometric analysis of PD-L1 expression on untreated HT-29 cancer cells cultured for 48 h.

Table S6. Region analysis – calculations as resulted from the CyFlow® Partec Report software.

Region	Gate	Ungated	Count	Count/mL	%Gated	Mean-x	CV-x%	Mean-y	CV-y%
R1	<None>	7454	7454	-	72.54	3.21	58.94	16.89	40.95
RN1	G1	9513	4681	-	91.03	**19.56**	104.78	-	-
R2	<None>	5277	5277	-	51.35	1.05	364.10	0.26	10.61

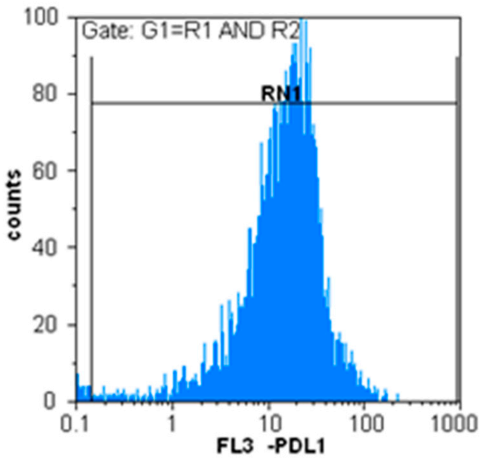

Figure S7. Flow cytometric analysis of PD-L1 expression on HT-29 cancer cells treated with KA39 at IC_{50} concentration (μM) for 48 h.

Table S7. Region analysis – calculations as resulted from the CyFlow® Partec Report software.

Region	Gate	Ungated	Count	Count/mL	%Gated	Mean-x	CV-x%	Mean-y	CV-y%
R1	<None>	7237	7237	-	69.64	4.31	59.31	15.58	40.30
RN1	G1	9855	4524	-	93.88	**20.13**	92.52	-	-
R2	<None>	4908	4908	-	47.23	0.61	424.39	0.26	9.36

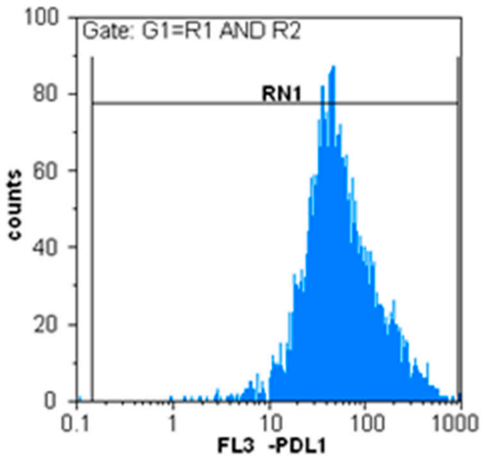

Figure S8. Flow cytometric analysis of PD-L1 expression on HT-29 cancer cells treated with KA39 at IC_{50} concentration (µM) for 72 h.

Table S8. Region analysis – calculations as resulted from the CyFlow® Partec Report software.

Region	Gate	Ungated	Count	Count/mL	%Gated	Mean-x	CV-x%	Mean-y	CV-y%
R1	<None>	6746	6746	-	64.78	7.51	62.31	13.81	39.76
RN1	G1	10280	3698	-	99.76	**80.82**	108.17	-	-
R2	<None>	3752	3752	-	36.03	0.71	513.16	0.25	8.38

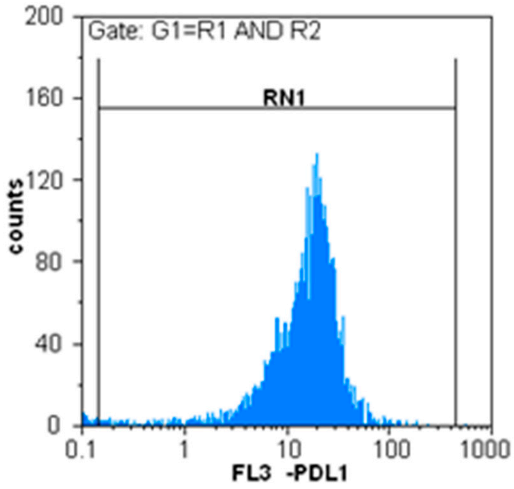

Figure S9. Flow cytometric analysis of PD-L1 expression on untreated HT-29 cancer cells cultured for 72 h.

Table S9. Region analysis – calculations as resulted from the CyFlow® Partec Report software.

Region	Gate	Ungated	Count	Count/mL	%Gated	Mean-x	CV-x%	Mean-y	CV-y%
R1	<None>	10905	10905	-	53.67	3.14	51.40	16.50	35.98
RN1	G1	19242	4057	-	95.28	**19.40**	75.77	-	-
R2	<None>	4516	4516	-	22.22	0.93	361.94	0.27	8.62

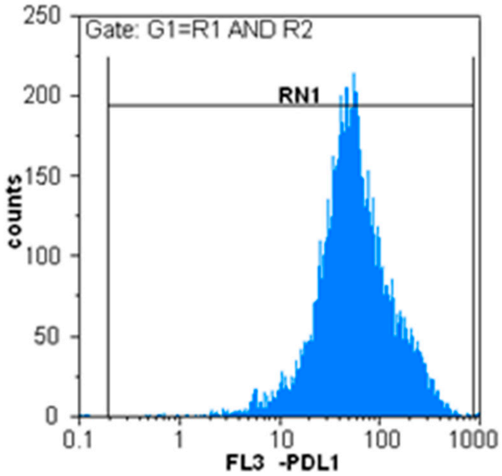

Figure S10. Flow cytometric analysis of PD-L1 expression on HT-29 cancer cells treated with KA39 at TGI concentration (μM) for 72 h**.**

Table S10. Region analysis – calculations as resulted from the CyFlow® Partec Report software.

Region	Gate	Ungated	Count	Count/mL	%Gated	Mean-x	CV-x%	Mean-y	CV-y%
R1	<None>	15069	15069	-	59.69	6.62	51.50	13.82	35.37
RN1	G1	24557	9083	-	99.67	**78.56**	95.78	-	-
R2	<None>	9409	9409	-	37.27	0.38	507.46	0.25	8.12

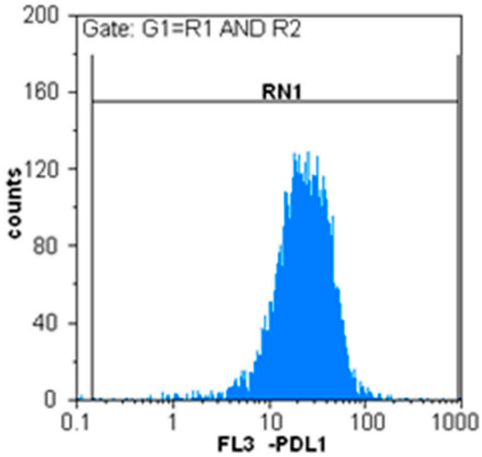

Figure S11. Flow cytometric analysis of PD-L1 expression on untreated DU-145 cancer cells cultured for 48 h.

Table S11. Region analysis – calculations as resulted from the CyFlow® Partec Report software.

Region	Gate	Ungated	Count	Count/mL	%Gated	Mean-x	CV-x%	Mean-y	CV-y%
R1	<None>	6999	6999	-	68.60	3.68	55.48	18.82	36.42
RN1	G1	9957	5387	-	99.57	**27.99**	79.41	-	-
R2	<None>	5591	5591	-	54.80	1.08	403.67	0.26	10.67

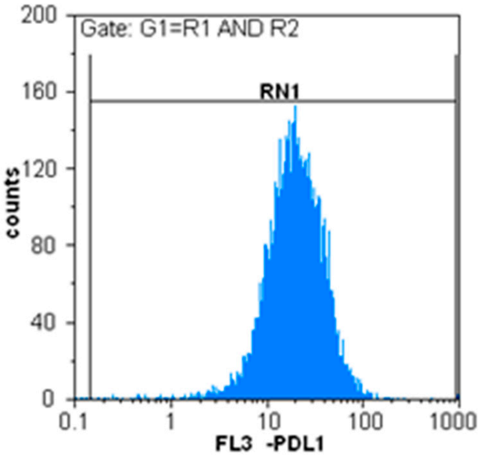

Figure S12. Flow cytometric analysis of PD-L1 expression on DU-145 cancer cells treated with KA39 at IC_{50} concentration (μM) for 48 h.

Table S12. Region analysis – calculations as resulted from the CyFlow® Partec Report software.

Region	Gate	Ungated	Count	Count/mL	%Gated	Mean-x	CV-x%	Mean-y	CV-y%
R1	<None>	7513	7513	-	71.72	3.43	49.02	18.89	32.36
RN1	G1	10201	6007	-	99.29	**24.48**	77.58	-	-
R2	<None>	6175	6175	-	58.95	0.75	419.37	0.26	9.44

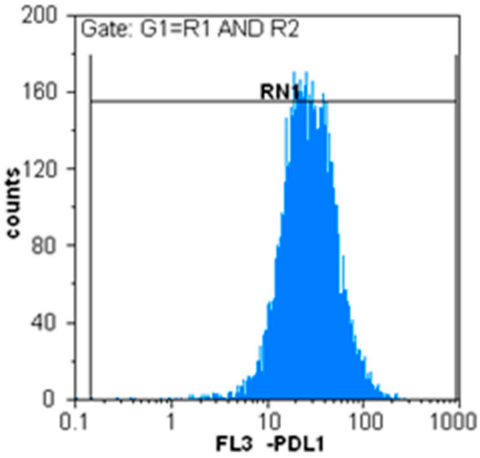

Figure S13. Flow cytometric analysis of PD-L1 expression on DU-145 cancer cells treated with KA39 at IC_{50} concentration (μM) for 72 h.

Table S13. Region analysis – calculations as resulted from the CyFlow® Partec Report software.

Region	Gate	Ungated	Count	Count/mL	%Gated	Mean-x	CV-x%	Mean-y	CV-y%
R1	<None>	8257	8257	-	75.15	5.15	55.46	21.17	35.94
RN1	G1	10802	7069	-	99.80	**33.55**	69.66	-	-
R2	<None>	7199	7199	-	65.52	1.50	387.07	0.27	11.54

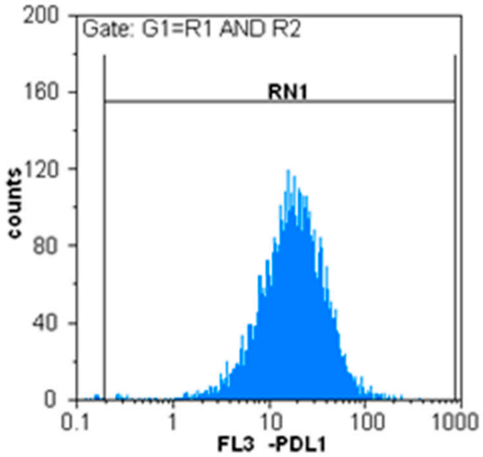

Figure S14. Flow cytometric analysis of PD-L1 expression on untreated DU-145 cancer cells cultured for 72 h.

Table S14. Region analysis – calculations as resulted from the CyFlow® Partec Report software.

Region	Gate	Ungated	Count	Count/mL	%Gated	Mean-x	CV-x%	Mean-y	CV-y%
R1	<None>	6907	6907	-	34.03	3.59	61.76	16.72	39.44
RN1	G1	19285	5088	-	99.10	**23.46**	86.03	-	-
R2	<None>	5726	5726	-	28.21	0.74	444.21	0.26	9.41

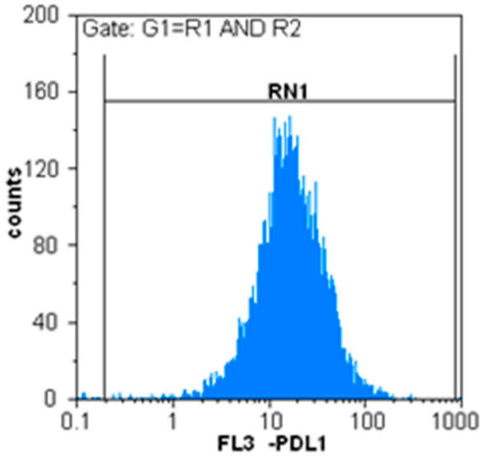

Figure S15. Flow cytometric analysis of PD-L1 expression on DU-145 cancer cells treated with KA39 at TGI concentration (μM) for 72 h.

Table S15. Region analysis – calculations as resulted from the CyFlow® Partec Report software.

Region	Gate	Ungated	Count	Count/mL	%Gated	Mean-x	CV-x%	Mean-y	CV-y%
R1	<None>	8572	8572	-	42.07	3.65	65.18	18.34	41.79
RN1	G1	19546	6736	-	99.44	**23.02**	94.70	-	-
R2	<None>	7311	7311	-	35.88	1.18	405.80	0.26	9.86

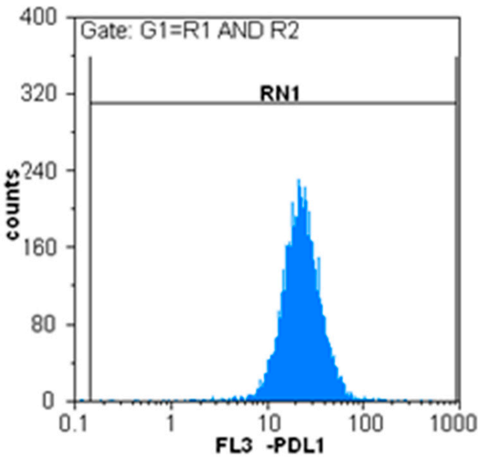

Figure S16. Flow cytometric analysis of PD-L1 expression on untreated PC-3 cancer cells cultured for 48 h.

Table S16. Region analysis – calculations as resulted from the CyFlow® Partec Report software.

Region	Gate	Ungated	Count	Count/mL	%Gated	Mean-x	CV-x%	Mean-y	CV-y%
R1	<None>	8410	8410	-	81.44	3.17	55.75	16.59	32.58
RN1	G1	10263	6134	-	99.89	**25.91**	64.58	-	-
R2	<None>	6212	6212	-	60.16	0.35	521.31	0.25	6.99

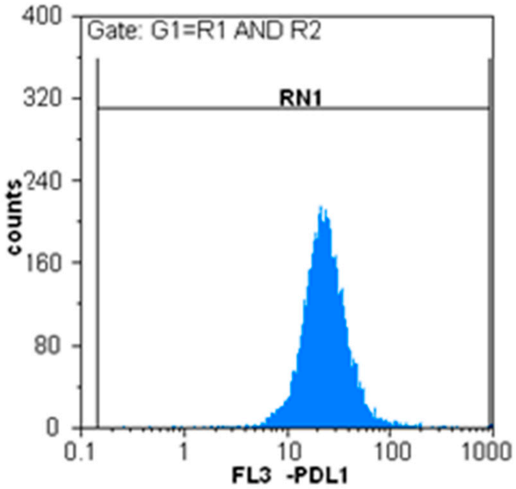

Figure S17. Flow cytometric analysis of PD-L1 expression on PC-3 cancer cells treated with KA39 at IC_{50} concentration (μM) for 48 h**.**

Table S17. Region analysis – calculations as resulted from the CyFlow® Partec Report software.

Region	Gate	Ungated	Count	Count/mL	%Gated	Mean-x	CV-x%	Mean-y	CV-y%
R1	<None>	8404	8404	-	80.95	4.19	61.38	17.63	34.04
RN1	G1	10257	6087	-	99.90	**27.66**	83.06	-	-
R2	<None>	6169	6169	-	59.42	0.42	444.45	0.25	7.22

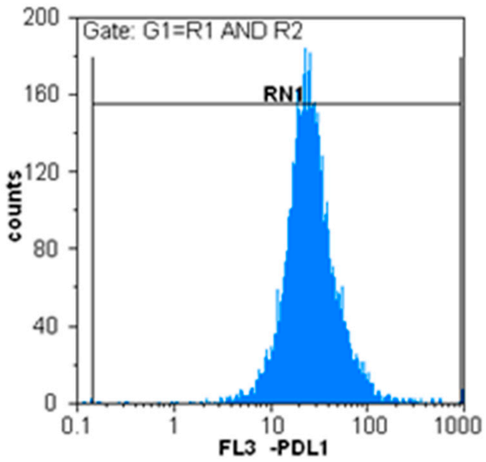

Figure S18. Flow cytometric analysis of PD-L1 expression on PC-3 cancer cells treated with KA39 at IC_{50} concentration (μM) for 72 h.

Table S18. Region analysis – calculations as resulted from the CyFlow® Partec Report software.

Region	Gate	Ungated	Count	Count/mL	%Gated	Mean-x	CV-x%	Mean-y	CV-y%
R1	<None>	8381	8381	-	80.02	6.37	53.97	16.00	36.76
RN1	G1	10305	5663	-	99.68	**32.98**	108.90	-	-
R2	<None>	5751	5751	-	54.91	0.34	475.01	0.25	7.03

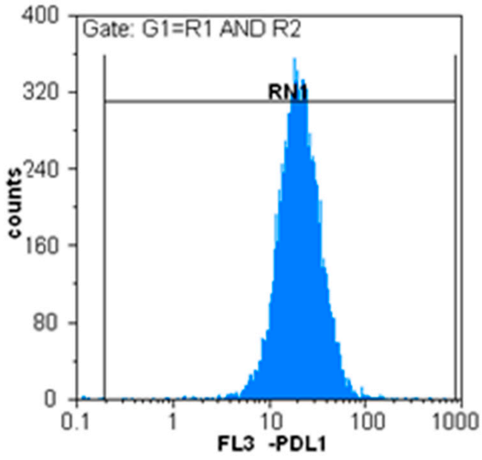

Figure S19. Flow cytometric analysis of PD-L1 expression on untreated PC-3 cancer cells cultured for 72 h.

Table S19. Region analysis – calculations as resulted from the CyFlow® Partec Report software.

Region	Gate	Ungated	Count	Count/mL	%Gated	Mean-x	CV-x%	Mean-y	CV-y%
R1	<None>	14475	14475	-	61.79	3.23	47.03	17.16	27.35
RN1	G1	22965	10842	-	99.83	**23.93**	69.66	-	-
R2	<None>	11321	11321	-	48.33	0.33	460.49	0.25	6.47

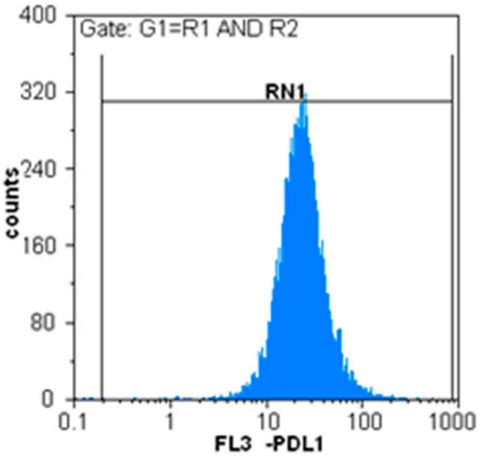

Figure S20. Flow cytometric analysis of PD-L1 expression on PC-3 cancer cells treated with KA39 at TGI concentration (μM) for 72 h.

Table S20. Region analysis – calculations as resulted from the CyFlow® Partec Report software.

Region	Gate	Ungated	Count	Count/mL	%Gated	Mean-x	CV-x%	Mean-y	CV-y%
R1	<None>	12827	12827	-	61.02	5.46	51.65	17.35	33.49
RN1	G1	20598	9903	-	99.84	**28.21**	74.87	-	-
R2	<None>	10297	10297	-	48.98	0.36	469.83	0.26	7.40

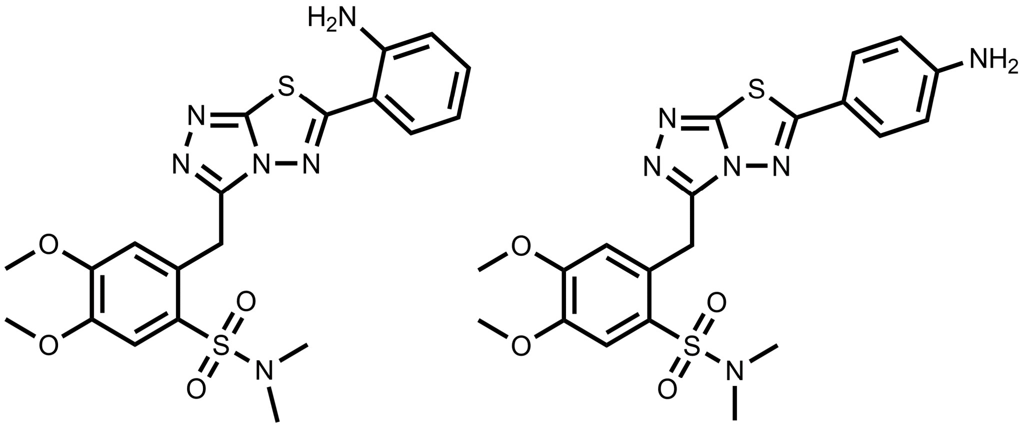

XK57 triazolo[3,4-b] thiadiazol derivative XK71 triazolo[3,4-b] thiadiazol derivative

Figure S21. Chemical structures of XK 57: 2-((6-(2-aminophenyl)-[1,2,4]triazolo[3,4-b][1,3,4]thiadiazol-3-yl)methyl)-4,5-dimethoxy-*N*,*N*-dimethylbenzenesulfonamide and XK 71: 2-((6-(4-aminophenyl)-[1,2,4]triazolo[3,4-b][1,3,4]thiadiazol-3-yl)methyl)-4,5-dimethoxy-*N*,*N*-dimethylbenzenesulfonamide.

Table S21. Cytostatic (GI_{50}, TGI) and cytotoxic effects (IC_{50}) induced by XK57 and XK71 on the tested human cancer cell lines, DLD-1, HT-29, LS174T, DU-145 and PC-3.

Cancer Cell Lines	XK57 GI_{50}	XK57 TGI	XK57 IC_{50}	XK71 GI_{50}	XK71 TGI	XK71 IC_{50}
DLD-1	90 μM	>100 μM	>100 μM	>100 μM	>100 μM	>100 μM
HT-29	>100 μM	>100 μM	>100 μM	>100 μM	>100 μM	>100 μM
LS174T	>100 μM	>100 μM	>100 μM	>100 μM	>100 μM	>100 μM
DU-145	>100 μM	>100 μM	>100 μM	>100 μM	>100 μM	>100 μM
PC-3	>100 μM	>100 μM	>100 μM	>100 μM	>100 μM	>100 μM

Table S22. The absolute values of PD-L1 expression as defined by flow cytometric analysis in untreated (control) and treated cells with XK57 derivative at IC_{50} concentration (μM) for 48 and 72 h, as well as at TGI concentration (μM) for 72 h. The units of measurements are expressed as mean of immuno-fluorescence intensity counted per cell.

Cancer cell lines	PD-L1 Expression				
	Control 48 h	XK57 IC_{50} 48 h	XK57 IC_{50} 72 h	Control 72 h	XK57 TGI 72 h
DLD-1	16.89 ± 0.84	19.88 ± 0.99	15.77 ± 0.94	25.09 ± 1.25	26.54 ± 1.59
HT-29	23.34 ± 1.4	24.79 ± 1.98	26.34 ± 1.31	21.23 ± 2.12	23.19 ± 1.62
DU-145	28.89 ± 2.02	27.19 ± 2.17	32.09 ± 2.24	24.56 ± 1.96	25.18 ± 1.25
PC-3	27.14 ± 2.44	28.44 ± 1.42	29.08 ± 2.03	27.88 ± 1.39	29.67 ± 2.07

Table S23. The absolute values of PD-L1 expression as defined by flow cytometric analysis in untreated (control) and treated cells with XK71 derivative at IC_{50} concentration (μM) for 48 and 72 h, as well as at TGI concentration (μM) for 72 h. The units of measurements are expressed as mean of immuno-fluorescence intensity counted per cell.

Cancer cell lines	PD-L1 expression				
	Control 48 h	XK71 IC_{50} 48 h	XK71 IC_{50} 72 h	Control 72 h	XK71 TGI 72 h
DLD-1	14.09 ± 0.7	17.45 ± 1.04	13.43 ± 0.67	22.81 ± 1.14	21.19 ± 1.05
HT-29	22.78 ± 1.36	21.31 ± 1.06	27.32 ± 1.63	20.59 ± 1.23	24.64 ± 1.47
DU-145	25.35 ± 1.26	27.53 ± 1.65	28.77 ± 2.01	26.89 ± 2.15	25.04 ± 1.75
PC-3	28.19 ± 1.97	30.98 ± 2.78	26.19 ± 1.83	25.12 ± 1.75	27.23 ± 2.17

MSI analysis in DLD-1, HT-29, DU-145, PC-3 and LS174T cancer cells treated with XK71 and XK57 derivatives

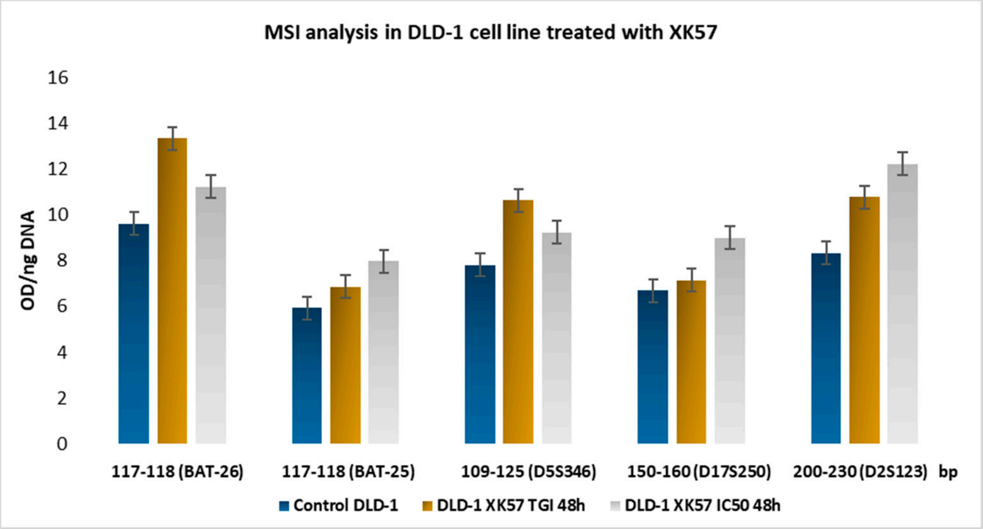

(**A**)

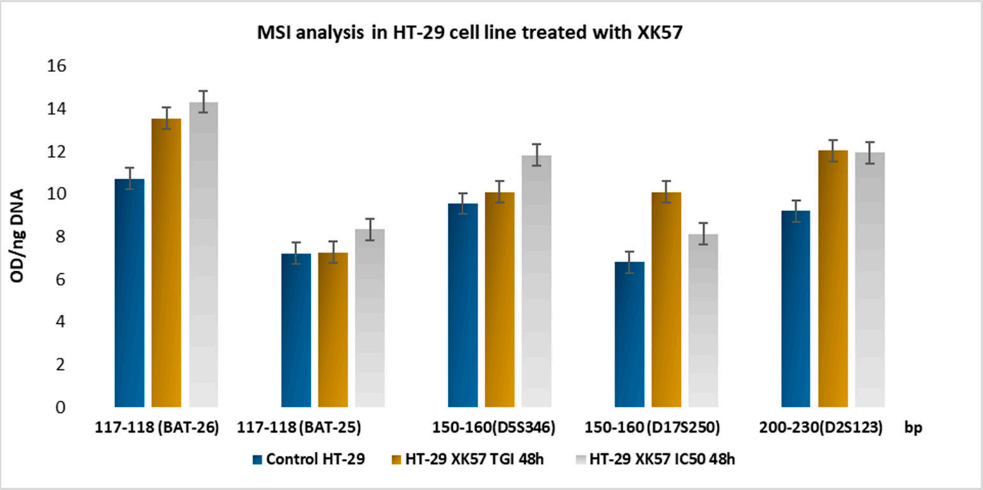

(**B**)

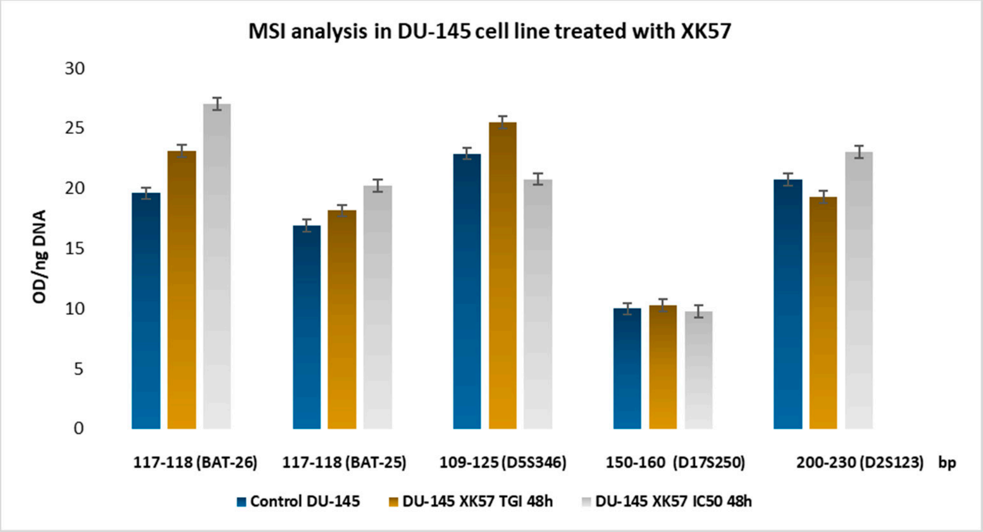

(**C**)

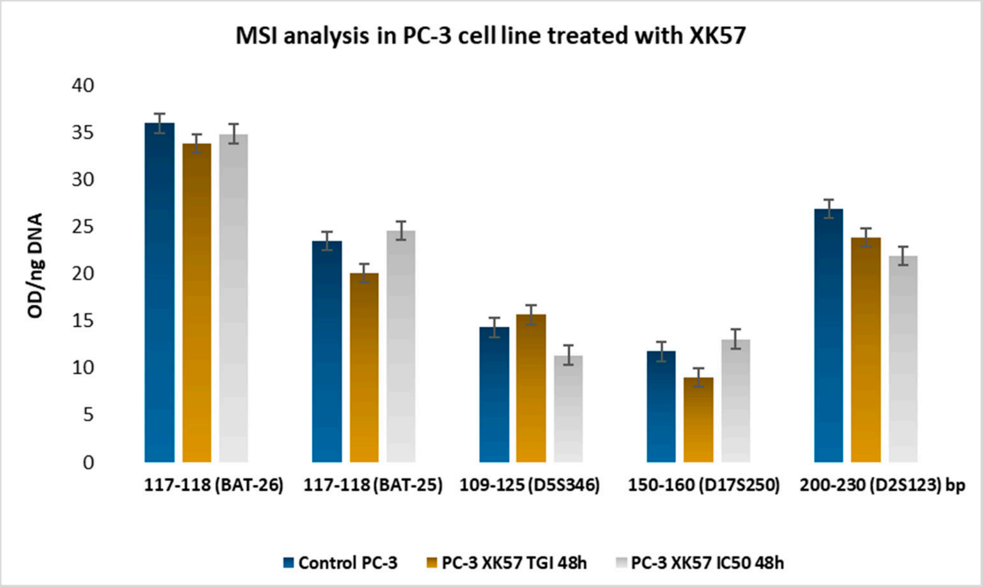

(**D**)

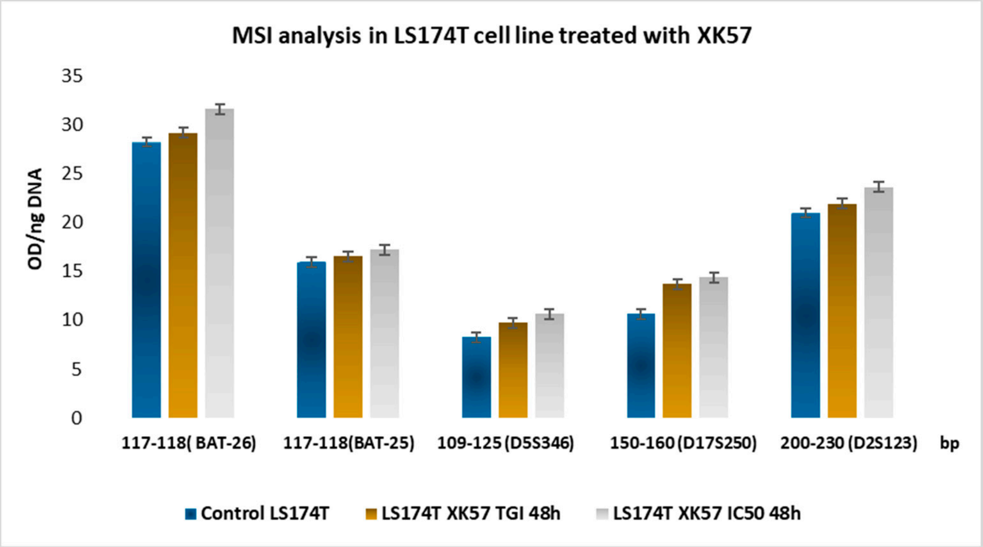

(**E**)

Figure S22. MSI alterations (mean ± SEM) induced by XK57 triazolo[3,4-b]thiadiazole derivative (per ng DNA) in five human cancer cell lines. (**A**), (**B**), (**C**), (**D**) and (**E**) demonstrate the MSI alterations in DLD-1, HT-29, DU-145, PC-3, and LS174T cancer cells, treated with XK57 at the TGI concentration and IC_{50} (μM) for 48h, respectively. Quantitative MSI fragments analysis conducted in Bethesda panel (BAT-26, BAT-25, D5S346, D17S250 and D2S123). Student t-test; $p > 0.01$; no statistical significance level.

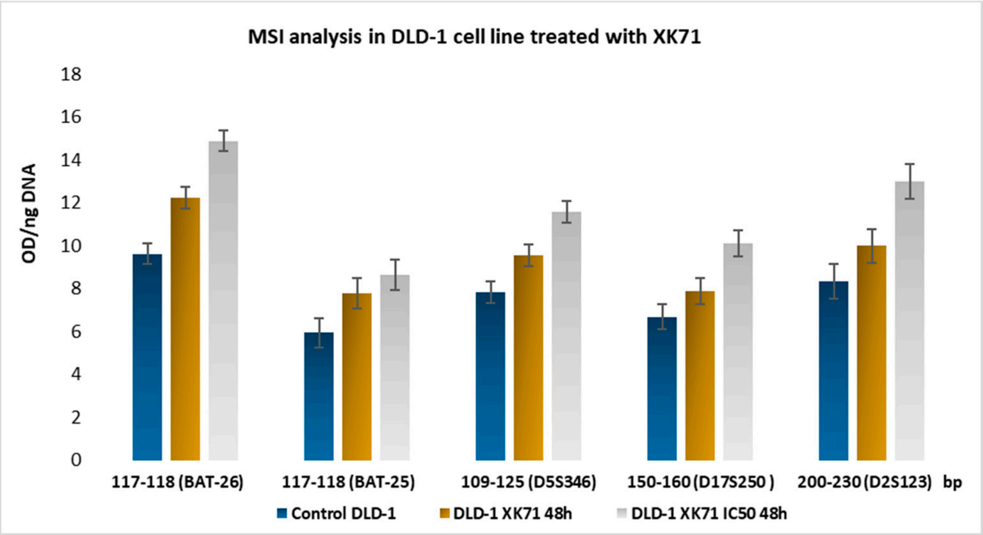
MSI analysis in DLD-1 cell line treated with XK71
OD/ng DNA
18
16
14
12
10
8
6
4
2
0
117-118 (BAT-26)
117-118 (BAT-25)
109-125 (D5S346)
150-160 (D17S250)
200-230 (D2S123)
bp
Control DLD-1
DLD-1 XK71 48h
DLD-1 XK71 IC50 48h

(**A**)

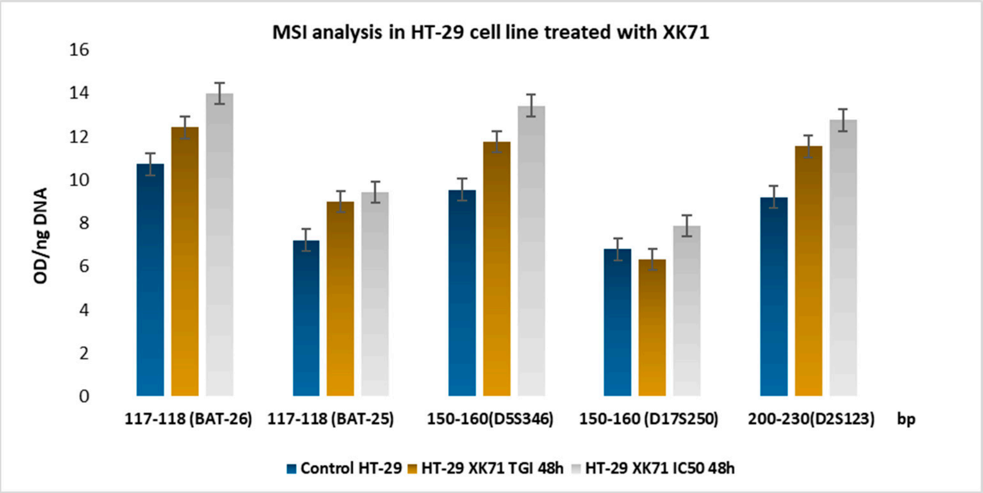
MSI analysis in HT-29 cell line treated with XK71
OD/ng DNA
16
14
12
10
8
6
4
2
0
117-118 (BAT-26)
117-118 (BAT-25)
150-160(D5S346)
150-160 (D17S250)
200-230(D2S123)
bp
Control HT-29
HT-29 XK71 TGI 48h
HT-29 XK71 IC50 48h

(**B**)

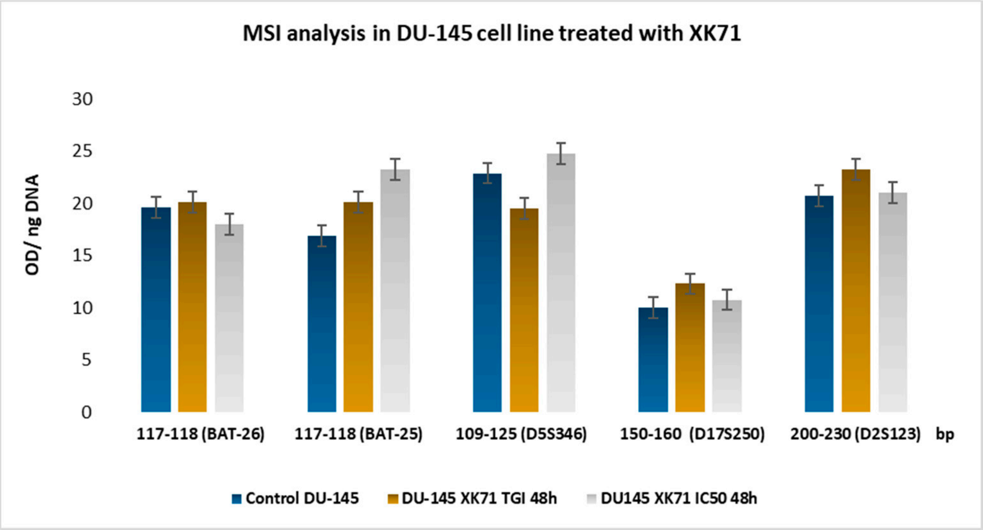
MSI analysis in DU-145 cell line treated with XK71
OD/ ng DNA
30
25
20
15
10
5
0
117-118 (BAT-26)
117-118 (BAT-25)
109-125 (D5S346)
150-160 (D17S250)
200-230 (D2S123)
bp
Control DU-145
DU-145 XK71 TGI 48h
DU145 XK71 IC50 48h

(**C**)

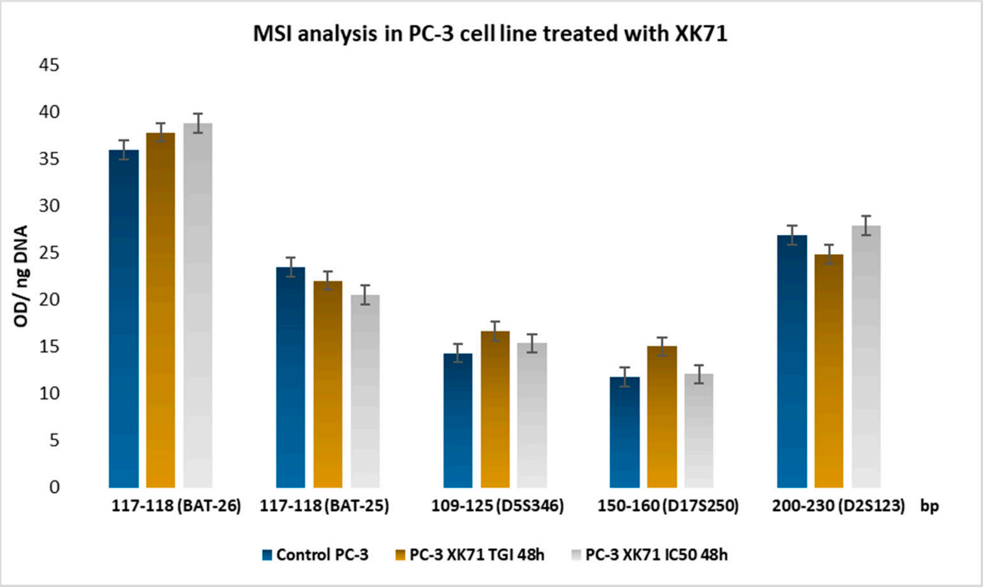

(**D**)

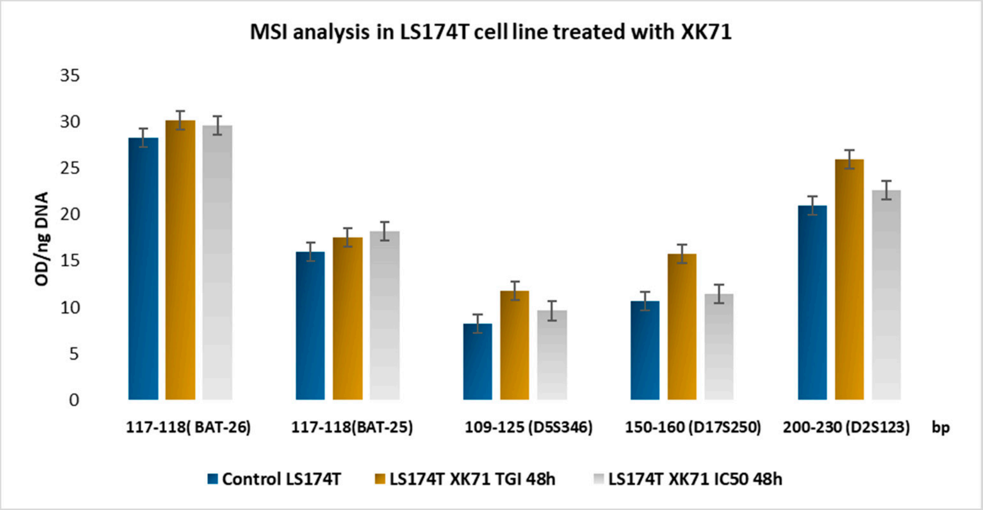

(**E**)

Figure S23. MSI alterations (mean ± SEM) induced by XK71 triazolo[3,4-*b*]thiadiazole derivative (per ng DNA) in five human cancer cell lines. (**A**), (**B**), (**C**), (**D**) and (**E**) show the MSI alterations in DLD-1, HT-29, DU-145, PC-3, and LS174T cancer cells, treated with XK71 at the TGI concentration and IC_{50} (μM) for 48h, respectively. Quantitative MSI fragments analysis conducted in Bethesda panel (BAT-26, BAT-25, D5S346, D17S250 and D2S123). Student t-test; $p > 0.01$; no statistical significance level.
